# Quercetin-1,2,3-Triazole Hybrids as Multifunctional Anti-Alzheimer’s Agents

**DOI:** 10.3390/molecules28227495

**Published:** 2023-11-09

**Authors:** Elisabete P. Carreiro, Ana R. Costa, Célia M. Antunes, Sofia Ernesto, Flávia Pinto, Beatriz Rodrigues, Anthony J. Burke

**Affiliations:** 1 Institute for Research and Advanced Training (IIFA), LAQV-REQUIMTE, University of Évora, Rua Romão Ramalho 59, 7000-671 Évora, Portugal; 2 Department of Medical and Health Sciences, School of Health and Human Development, University of Évora, Rua Romão Ramalho 59, 7000-671 Évora, Portugal; 3 Institute of Earth Sciences, Institute of Research and Advanced Training, University of Évora, 7000-671 Évora, Portugal; 4 Academic Clinical Center of Alentejo, C-TRAIL, Rua Romão Ramalho 59, 7000-671 Évora, Portugal; 5 Department of Chemistry and Biochemistry, School of Sciences and Technologies, University of Évora, Rua Romão Ramalho 59, 7000-671 Évora, Portugal; 6 Faculty Pharmacy, University of Coimbra, Pólo das Ciências da Saúde, Azinhaga de Santa Comba, 3000-548 Coimbra, Portugal

**Keywords:** quercetin, 1,2,3-triazole, hybrids, Alzheimer’s diseases, cholinesterase inhibitory assays, antioxidant assay, toxicity, ROS protection

## Abstract

The number of patients with Alzheimer’s disease (AD) continues to rise and, despite the efforts of researchers, there are still no effective treatments for this multifaceted disease. The main objective of this work was the search for multifunctional and more effective anti-Alzheimer agents. Herein, we report the evaluation of a library of quercetin-1,2,3-triazole hybrids (**I–IV**) in antioxidant, hydrogen peroxide-induced oxidative stress protection, and cholinesterases (AChE and BuChE) inhibitory activities. Hybrids **IIf** and **IVa-d** showed potent in vitro inhibitory activity on *eq*BuChE (IC_50_ values between 11.2 and 65.7 μM). Hybrid **IIf**, the best inhibitor, was stronger than galantamine, displaying an IC_50_ value of 11.2 μM for *eq*BuChE, and is also a competitive inhibitor. Moreover, toxicity evaluation for the most promising hybrids was performed using the *Artemia salina* toxicity assay, showing low toxicity. Hybrids **IIf**, **IVb,** and **IVd** did not affect viability at 12.5 μM and also displayed a protective effect against oxidative stress induced by hydrogen peroxide in cell damage in MCF-7 cells. Hybrids **IIf**, **IVb,** and **IVd** act as multifunctional ligands in AD pathologies.

## 1. Introduction

Alzheimer’s disease (AD) is a neurodegenerative disease with high mortality, for which there is still no cure, despite all the efforts of researchers in the last three decades [1]. AD is undoubtedly a complex and multifactorial disease, and its etiology remains elusive. Various hypotheses have been described as being responsible for the development of this pathology, such as cholinergic dysfunction [2], aggregation of the β-amyloid peptide [3], accumulation of hyperphosphorylated tau protein [4], dyshomeostasis of biomaterials [5], oxidative stress [6,7], mitochondrial dysfunction [8], and neuroinflammation [9,10,11]. Perhaps this complexity is the reason why we still have not found a cure for this terrible disease, whose number of patients is expected to reach 153 million by 2050. However, many researchers have begun to develop new compounds that can act on different molecular targets associated with this pathology [12].

Flavonoids, a class of polyphenols present in our diet, possess multiple biological activities, including anti-AD effects. However, flavonoids have some limitations, such as low bioavailability and permeability, which compromise their therapeutic efficacy [13]. Quercetin, one of the most abundant flavonoids in plants and in our diet, has been shown to have strong potential in the combat of Alzheimer’s disease [14,15]. Quercetin can act on different general mechanisms of AD etiology in a variety of in vitro and in vivo models, such as protecting neuronal cells by attenuating oxidative stress and neuroinflammation [16,17], inhibiting Aβ aggregation and tau phosphorylation [18,19], and restoring cholinergic function (acetylcholinesterase (AChE) and butyrylcholinesterase (BuChE) inhibition) [20]. However, its clinical efficacy needs to be improved by derivatizing its structure in order to increase bioavailability and greater intestinal and brain penetrability.

Molecules containing the 1,2,3-triazole heterocyclic in their structure also have a wide range of pharmacological properties, including anti-AD [21]. To improve the therapeutic efficacy of quercetin, it was decided to combine it with the 1,2,3-triazole heterocyclic unit through a molecular-hybridization approach. These hybrids also have a greater potential to act as multifunctional molecules to combat Alzheimer’s disease, which was our goal.

**Figure 1:**
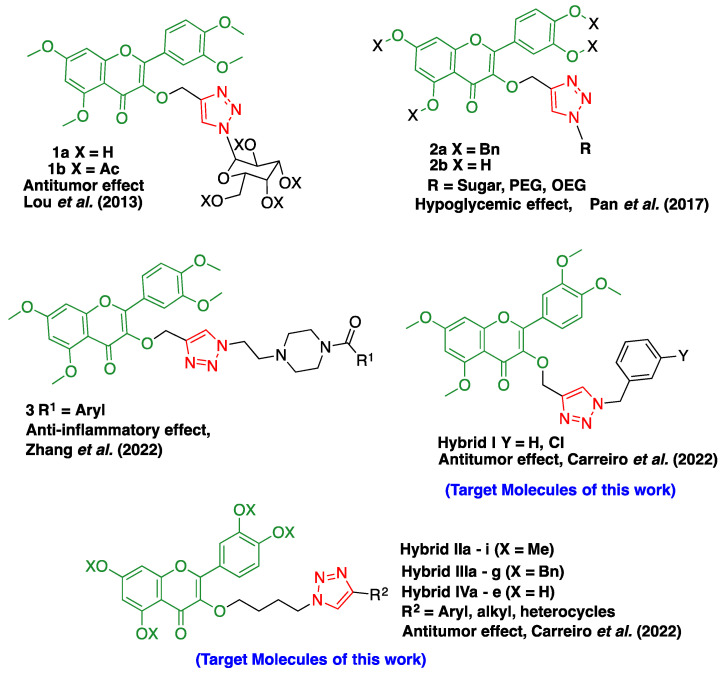
Quercetin-1,2,3-triazole hybrids reported in the literature with antitumor, anti-inflammatory, and hypoglycemic effects [23,24,25,26].

In the literature, a wide variety of flavonoid-1,2,3-triazole hybrids are known to exhibit a wide range of biological properties, such as antitumor, antimicrobial, antidiabetic, neuroprotective, anti-inflammatory, and antioxidant properties, among others [22]. However, only a few examples of quercetin derivatives functionalized with a 1,2,3-triazole heterocyclic in the 3-OH position are reported; see Figure 1 [23,24,25,26].

**Scheme 1:**
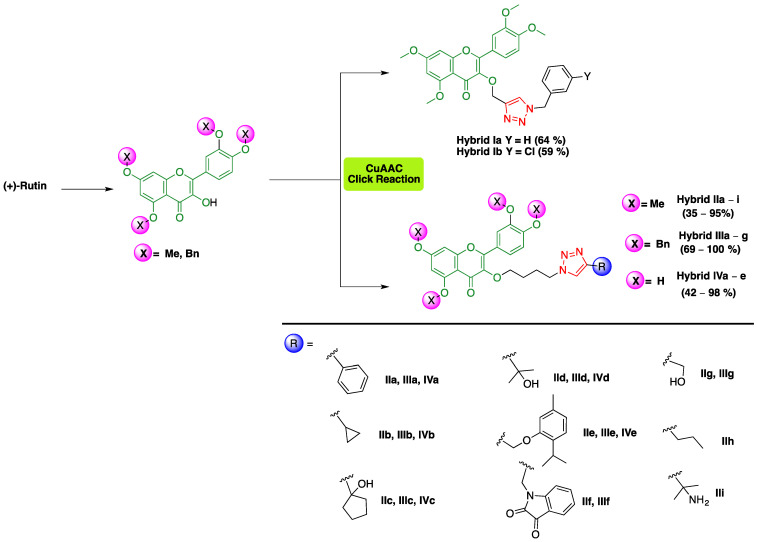
Quercetin-1,2,3-triazole hybrids studied in this work [26].

The quercetin-1,2,3-triazole hybrids **I–IV** developed by our group in 2022 were prepared by functionalizing the 3-OH of quercetin with a short alkyl chain and then with a 1,2,3-triazole moiety through copper-catalyzed azide-alkyne cycloaddition (CuAAC), the “click” reaction. Four types of hybrid were prepared, the difference between them being the protection of the remaining hydroxyl groups of quercetin with a methyl group (in this case hybrids **I** and **II**) or a benzyl group (hybrids **III**). Hybrids **IV** have the hydroxyl groups of quercetin free, and were prepared via the debenzylation of hybrids **III** [26]. The protection of the hydroxyl groups can make the hybrids more permeable to membranes [27]. These interesting hybrids were previously prepared and fully characterized, and their antiproliferative activity was evaluated on the REM-138 cell line (canine breast cancer) [26]. The most active hybrids exhibited extraordinary IC_50_ values, with the lowest value of only 41 nM. These chemical modifications in the structure of the quercetin moiety were crucial to increasing the antiproliferative activity in the cancer cell line. The synthetic route used is shown in Scheme 1.

To evaluate the multifunctional behavior of our quercetin-1,2,3-triazole hybrids **I–IV** (Scheme 1) as anti-AD agents, these compounds were screened against AChE and BuChE and also for antioxidant activities. Their protective effect against oxidative stress induced by hydrogen peroxide in cell damage and toxicity was also assessed.

## 2. Results and Discussion

### 2.1. Pharmacological Evaluation

#### 2.1.1. Cholinesterases (ChEs) Inhibitory Activity

The cholinergic function of patients with Alzheimer’s disease can be improved by inhibiting cholinesterases (AChE and BuChE), which leads to an increase in the level of acetylcholine, leading to greater communication between nerve cells. Given its importance in the treatment of AD, there is great interest in finding a new cholinesterase inhibitor with superior efficacy and fewer side effects than the drugs currently used (galantamine, donepezil and rivastigmine).

Firstly, the inhibitory activity of the quercetin-1,2,3-triazole hybrids **I**–**IV** was screened in vitro on *ee*AChE and *eq*BuChE at a concentration of 100 μM (these enzymes are considered as models of the human isoform [28]). The ChE inhibitory activity of all compounds was evaluated using the method of Ellman [29]. Galantamine and quercetin were used as reference compounds in this assay. The preliminary results are listed in Table 1. In general, the tested hybrids showed poor inhibition of *ee*AChE (Table 1), inferior to 40.6%. On the other hand, most of these hybrids were effective for *eq*BuChE inhibition (Table 1). In particular, hybrids **I**, **II,** and **III** showed low to moderate % inhibition, unlike hybrids **IV**, which showed very good to excellent values (Table 1). Hybrids **IIIb**, **e,** and **g** were insoluble.

#### 2.1.2. BuChE Inhibitory Assay

**Figure 2:**
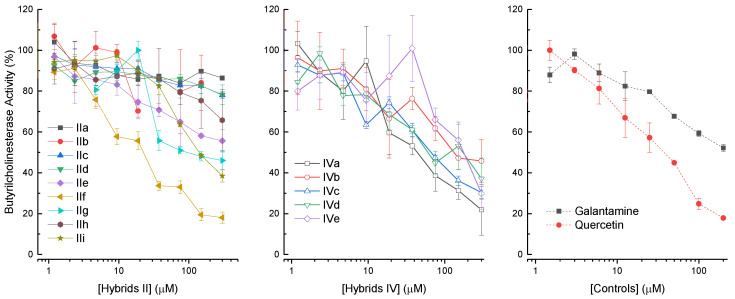
Inhibitory effects of hybrids **II** and **IV** on *eq*BuChE activity. Values are expressed as the mean ± sd (experiments performed in triplicate). Galantamine and quercetin served as positive controls.

**Figure 3:**
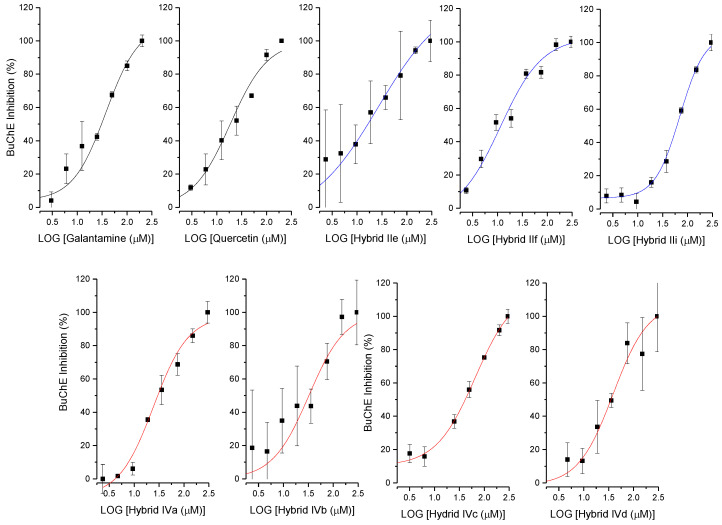
Dose–response curve plots of inhibition percentage for hybrids **IIe**, **IIf**, **IIi**, **IVa**–**d**; quercetin; and galantamine.

Based on the screening results, only the hybrids **IIa–i**, **IVa–e** were selected to determine the IC_50_ for *eq*BuChE and were assayed in the concentration range of 1.2 and 300 μM. The results are shown in Figure 2 and Figure 3 and Table 2. Analyzing the results in Figure 2, we can see that the inhibitory effect of hybrids **IIa–i** on *eq*BuChE activity is more pronounced for hybrid **IIf**. In the case of hybrids **IVa–e**, the inhibitory effect is more accentuated than that of the majority of hybrids **II** (with the exception of hybrid **IIf**). The structural difference between hybrids **II** and **IV** is the protection of quercetin’s hydroxyls with methyl groups, which seems to affect the inhibitory potential. However, hybrid **IIf** is the best inhibitor despite being a methylated compound, but it has an isatin unit attached to the 1,2,3-triazole ring, which is probably relevant for the observed inhibitory effect. In fact, the isatin unit seems to be relevant in the binding of compounds to various pharmacologic targets, namely, enzymes (like COX2, alpha-amylase, and CDK2 [30]).

Figure 3 shows the dose–response curves for the best inhibitors, from which it was possible to determine the IC_50_, as displayed in Table 2. Comparing the inhibitory effect of quercetin with that of galantamine, quercetin is more potent.

With regard to the IC_50_ values obtained (Table 2), hybrids **IIe** and **IIf** show values of 26.6 and 11.2 μM, respectively, being more potent inhibitors than galantamine (37.6 μM). The structures of these hybrids are similar; both have the hydroxyl groups of the quercetin methylated and differ only in the substituent at the 4-position of the 1,2,3-triazole ring, with hybrid **IIe** having a thymol and **IIf** an isatin as substituents. Hybrid **IIf** is also more potent than quercetin (18.2 μM). As already mentioned, the **IV** hybrids are more potent than **II** hybrids, with the exception of compounds **IIe** and **IIf**. Hybrids **IVa,b** and **IVd** have the most promising IC_50_ values: 24.7, 32.4, and 39.0 μM, respectively. Hybrid **IVa** is a more potent inhibitor than galantamine but slightly weaker than quercetin (18.2 μM). Comparing the IC_50_ values obtained for hybrids **IIe** (26.6 μM) and **IVe** (>150 μM), the first hybrid is a more potent inhibitor than its unmethylated derivative. The opposite behavior is observed for hybrids **IIa–d** and their unmethylated ones. Optimistically, both families of type **II** and **IV** hybrids are promising *eq*BuChE inhibitors.

#### 2.1.3. Kinetic Study for the Inhibition of *eq*BuChE

**Figure 4:**
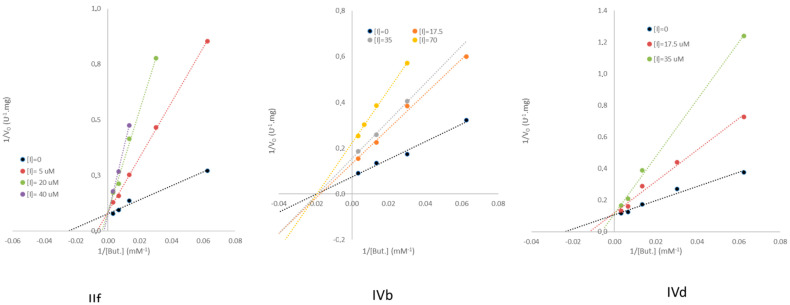
Kinetic study of the mechanism of *eq*BuChE inhibition by hybrids **IIf**, **IVb,** and **IVd**. Overlaid Lineweaver–Burk plots of the reciprocal of *eq*BuChEs initial velocity versus the reciprocal of substrate (BTCI) concentration, in the absence and in the presence of inhibitors (inhibitor concentrations expressed in µM).

Kinetic studies were performed to understand the mechanism of action of these hybrids on *eq*BuChE, hybrids **IIf, IVb,** and **IVd**, which showed very good to excellent inhibitory activity against *eq*BuChE. According to the Lineweaver–Burk plots (shown in Figure 4) and taking into account the kinetic parameters calculated from the linear regressions from Lineweaver–Burk analysis (Table 3), hybrids **IIf** and **IVd** showed competitive inhibition, whilst hybrid **IVb** was found to be the mixed type (non-competitive). The interaction with the active center of the enzyme thus seems to be favored by the presence of polar groups. Hybrid **IIf** presented the smaller K_i_ (Table 3), binding to the enzyme with the higher affinity, which can be relevant for pharmacologic applications in the future.

#### 2.1.4. Antioxidant Activity

**Figure 5:**
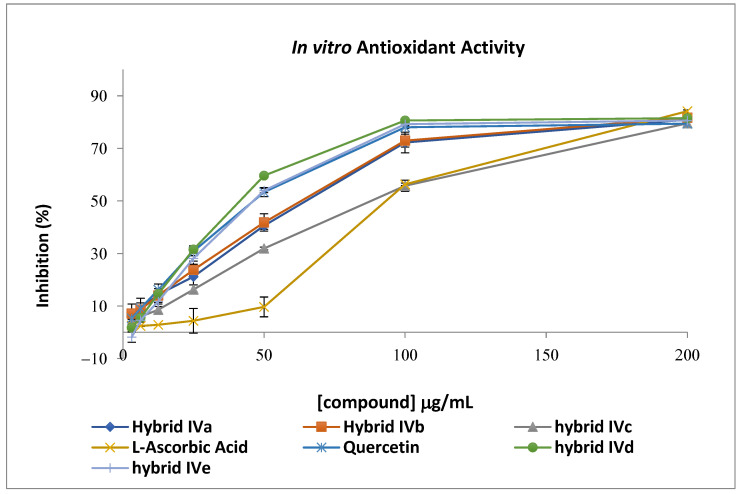
Determination of the DPPH radical scavenging activity % of inhibition vs. concentration of compounds is plotted.

To assess the antioxidant activity of hybrids **IVa–e**, **IIf** and quercetin, the 2,2-diphenyl-1-picrylhydrazine (DPPH) radical scavenging colorimetric method was used, which is one of the most widely used methods for determining antioxidant activity. In this method, antioxidants react with DPPH (which has a strong violet color) and convert it into yellow diphenylpicrylhydrazine. Compounds were assayed in the range of concentrations from 3 to 200 μg/mL. Ascorbic acid was used as positive control. The results of % of inhibition of free radicals obtained are presented in Figure 5. At a concentration of 100 μg/mL, hybrids **IVa,b**, **IVd–e** showed greater antioxidant activity than hybrid **IVb** and ascorbic acid, the positive control. Hybrid **IIf** showed no antioxidant activity in this concentration range. We can conclude that the free hydroxyl groups of quercetin are mainly responsible for inhibiting the free radicals since, in the case of hybrid **IIf,** the hydroxyl groups are methylated, which did not show any antioxidant activity. Another piece of evidence is the similarity between the % inhibition values of hybrids **IVa**,**b** and **IVd,e** and quercetin. In conclusion, the functionalization of quercetin with the 1,2,3-triazole unit did not affect its antioxidant activity, which means that the new quercetin hybrids also have strong antioxidant activity.

#### 2.1.5. Cellular Viability and Antioxidant Protection

**Figure 6:**
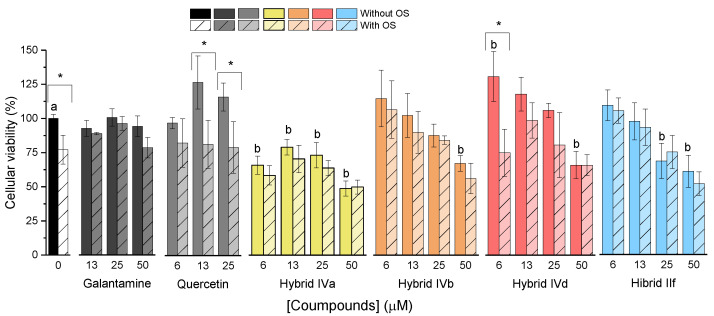
Effect of different concentrations of hybrids **IVa**, **IVb**, **IVd**, **IIf**, galantamine, and quercetin on cell viability in the presence and absence of an oxidative stressor (OS) (H_2_O_2_). MCF-7 cells were incubated with different concentrations of compounds (6, 13, 25, and 50 μM) for 72 h. The cell viability was measured using the WST-1 assay. Data are presented as means ± SD (* significant for *p* < 0.05).

Given the importance of oxidative stress-induced apoptosis of neuronal cells in AD, we present here a study to investigate the effect of quercetin-1,2,3-triazole hybrids on H_2_O_2_-induced oxidative damage in cultured MCF-7 cells, used as a human cell model. Viability was assessed with the WST-1 test in the absence and in the presence of an oxidative stressor (10^−4^ M H_2_O_2_). Quercetin and galantamine were included as positive controls. Hybrids were tested in the range of 6.25–50 μM; the results obtained are shown in Figure 6. Galantamine and quercetin did not reduce cellular viability in the concentrations tested. Hybrid **IVa** reduced cellular viability by 40% at 6.25 μM and should be excluded for anti-Alzheimer purposes. Hybrids **IVb**, **IVd,** and **IIf** did not affect viability at 12.5 μM. Cytotoxicity of **IVa** and **IVb** hybrids was previously tested in the REM cell line [26], although with an exposure period of 72 h. Hybrid **IVa** was revealed to be cytotoxic for REM cells >10 µM (72 h incubation) and also in our present study at 2 h of exposure, pointing to an underlying rapid cytotoxicity mechanism, suggesting an easy cellular penetration or membrane interaction. For Hybrid **IVb**, exposure time seems to be more relevant, being cytotoxic in REM cells >10 µM over 72 h incubation, but only >25 µM for MCF-7 with 2 h incubation time—but this difference could also be due to the different biological make-ups of the two cell lines.

As far as cellular antioxidant protection capacity is concerned, galantamine, unlike quercetin, showed good antioxidant cellular protection. Hybrids **IVb**, **IVd,** and **IIf** were equally effective in the antioxidant protection against the stressor H_2_O_2_. According to the results obtained, the hybrid compounds showed a better protective effect than quercetin, which leads us to conclude that the functionalization of quercetin with the 1,2,3-triazole heterocyclic can improve quercetin’s pharmacological potential, probably improving its cell-permeability capacity.

Interestingly, the hybrid **IIf**, which had not shown antioxidant capacity in the DPPH test (free radical mechanism of action), now shows a good amount of cellular antioxidant protection capacity. This could imply that its antioxidant mechanism of action may not involve free radicals scavaging, but another mechanism seems to be involved. Antioxidant properties associated with isatin units have already been reported [30].

#### 2.1.6. Toxicity Assays

**Figure 7:**
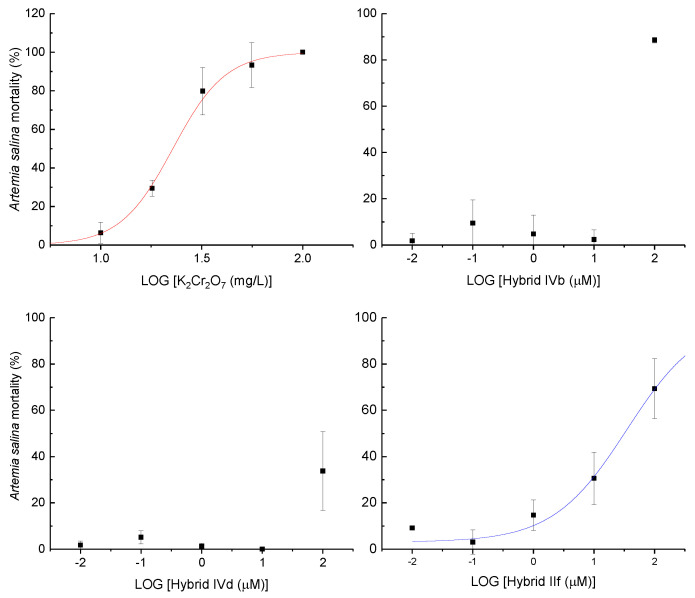
Mortality (%) of artemia salina vs. log[compound]—dose–response curves.

*Artemia salina* was used for in vivo general toxicity assessment of the promising hybrids **IVb**, **IVd,** and **IIf**; the results are shown in Table 4 and Figure 7. K_2_Cr_2_O_7_ was used as a control. Hybrid **IVd** presented the lowest toxicity. Hybrids **IVb** and **IIf** did not present toxicity in concentrations ≤10 μM.

A good correlation between the brine shrimp lethality assay and cytotoxicity potential is usually expected [31], although absorption, distribution, metabolism, and excretion must be taken into consideration when toxicologic studies are performed in living organisms. In our study, the results for the *Artemia salina* lethality assay are in good agreement with the cytotoxicity results for the same compounds, with hybrid **IVd** being the less-toxic compound (*A. salina* LC_50_ > 100 µM and cytotoxicity EC_50_ > 50 µM).

## 3. Materials and Methods

***General Remarks***

For the realization of this work, reagents were used as received. Iodide acetylthiocholine (ATCI), *S*-butyrylthiocholine iodide (BTCI) and 5,5′-dithiobis(2-nitrobenzoic acid) (DTNB) (Ellman’s reagent) were obtained from Sigma-Aldrich and Alfa Aesar. The selected enzymes were purchased from Sigma-Aldrich: electric eel acetylcholinesterase (*ee*AChE) (E.C.3.1.1.7, Type VI-S, lyophilized powder, 500 U/2 mg), and equine serum butyrylcholinesterase (*eq*BuChE) (E.C. 3.1.1.8, lyophilized powder, 10.9 U/mg). The commercial inhibitors used were galantamine (TCI) and quercetin (Sigma-Aldrich, St. Louis, MO, USA). 1,1-Diphenyl-2-picrylhydrazyl (DPPH), used to determine the radical-scavenging activity, was purchased from Alfa Aesar. All other reagents and solvents used in the experiments were of analytical grade and of the highest purity.

### 3.1. Quercetin-1,2,3-triazole Hybrids **I**–**IV**

Quercetin-1,2,3-triazole hybrids **I–IV** were already reported in literature by Carreiro et al. [26]. ^1^H spectra data are included in the Supplementary Information. The 23 hybrids evaluated were 3-((1-Benzyl-1*H*-1,2,3-triazol-4-yl)methoxy)-2-(3,4-dimethoxyphenyl)-5,7-dimethoxy-4*H*-chromen-4-one (**Ia**), 3-((1-(3-Chlorobenzyl)-1*H*-1,2,3-triazol-4-yl)methoxy)-2-(3,4-dimethoxyphenyl)-5,7-dimethoxy-4*H*-chromen-4-one (**Ib**); 2-(3,4-Dimethoxyphenyl)-5,7-dimethoxy-3-(4-(4-phenyl-1*H*-1,2,3-triazol-1-yl)butoxy)-4*H*-chromen-4-one (**IIa**); 2-(3,4-Dimethoxyphenyl)-3-(4-(4-cyclopropyl-*1H*-1,2,3-triazol-1-yl)butoxy)-5,7-dimethoxy-4*H*-chromen-4-one (**IIb**); 2-(3,4-Dimethoxyphenyl)-3-(4-(4-(1-hydroxycyclopentyl)-1*H*-1,2,3-triazol-1-yl)butoxy)-5,7-dimethoxy-4*H*-chromen-4-one (**IIc**); 2-(3,4-Dimethoxyphenyl)-3-(4-(4-(2-hydroxypropan-2-yl)-1*H*-1,2,3-triazol-1-yl)butoxy)-5,7-dimethoxy-4*H*-chromen-4-one (**IId**); 2-(3,4-Dimethoxyphenyl)-3-(4-(4-((2-isopropyl-5-methylphenoxy)methyl)-1*H*-1,2,3-triazol-1-yl)butoxy)-5,7-dimethoxy-4*H*-chromen-4-one (**IIe**); 1-((1-(4-((2-(3,4-Dimethoxyphenyl)-5,7-dimethoxy-4-oxo-4*H*-chromen-3-yl)oxy)butyl)-1*H*-1,2,3-triazol-4-yl)methyl)indoline-2,3-dione (**IIf**); 2-(3,4-Dimethoxyphenyl)-3-(4-(4-(hydroxymethyl)-1*H*-1,2,3-triazol-1-yl)butoxy)-5,7-dimethoxy-4*H*-chromen-4-one (**IIg**); 2-(3,4-Dimethoxyphenyl)-5,7-dimethoxy-3-(4-(4-propyl-1*H*-1,2,3-triazol-1-yl)butoxy)-4*H*-chromen-4-one (**IIh**); 3-(4-(4-(2-Aminopropan-2-yl)-1*H*-1,2,3-triazol-1-yl)butoxy)-2-(3,4-dimethoxyphenyl)-5,7-dimethoxy-4*H*-chromen-4-one (**IIi**), 5,7-Bis(benzyloxy)-2-(3,4-bis(benzyloxy)phenyl)-3-(4-(4-phenyl-1*H*-1,2,3-triazol-1-yl)butoxy)-4*H*-chromen-4-one (**IIIa**); 5,7-Bis(benzyloxy)-2-(3,4-bis(benzyloxy)phenyl)-3-(4-(4-cyclopropyl-1*H*-1,2,3-triazol-1-yl)butoxy)-4*H*-chromen-4-one (**IIIb**); 5,7-Bis(benzyloxy)-2-(3,4-bis(benzyloxy)phenyl)-3-(4-(4-(1-hydroxycyclopentyl)-1*H*-1,2,3-triazol-1-yl)butoxy)-4*H*-chromen-4-one (**IIIc**); 5,7-Bis(benzyloxy)-2-(3,4-bis(benzyloxy)phenyl)-3-(4-(4-(2-hydroxypropan-2-yl)-1*H*-1,2,3-triazol-1-yl)butoxy)-4*H*-chromen-4-one (**IIId**); 5,7-Bis(benzyloxy)-2-(3,4-bis(benzyloxy)phenyl)-3-(4-(4-((2-isopropyl-5-methylphenoxy)methyl)-1*H*-1,2,3-triazol-1-yl)butoxy)-4*H*-chromen-4-one (**IIIe**); 1-((1-(4-((5,7-Bis(benzyloxy)-2-(3,4-bis(benzyloxy)phenyl)-4-oxo-4*H*-chromen-3-yl)oxy)butyl)-1*H*-1,2,3-triazol-4-yl)methyl)indoline-2,3-dione (**IIIf**); 5,7-Bis(benzyloxy)-2-(3,4-bis(benzyloxy)phenyl)-3-(4-(4-(hydroxymethyl)-1*H*-1,2,3-triazol-1-yl)butoxy)-4*H*-chromen-4-one (**IIIg**); 2-(3,4-Dihydroxyphenyl)-5,7-dihydroxy-3-(4-(4-phenyl-1*H*-1,2,3-triazol-1-yl)butoxy)-4*H*-chromen-4-one (**IVa**); 3-(4-(4-Cyclopropyl-1*H*-1,2,3-triazol-1-yl)butoxy)-2-(3,4-dihydroxyphenyl)-5,7-dihydroxy-4*H*-chromen-4-one (**IVb**); 2-(3,4-Dihydroxyphenyl)-5,7-dihydroxy-3-(4-(4-(1-hydroxycyclopentyl)-1*H*-1,2,3-triazol-1-yl)butoxy)-4*H*-chromen-4-one (**IVc**); 2-(3,4-Dihydroxyphenyl)-5,7-dihydroxy-3-(4-(4-(2-hydroxypropan-2-yl)-1*H*-1,2,3-triazol-1-yl)butoxy)-4*H*-chromen-4-one (**IVd**); and 2-(3,4-Dihydroxyphenyl)-5,7-dihydroxy-3-(4-(4-((2-isopropyl-5-methylphenoxy)methyl)-1*H*-1,2,3-triazol-1-yl)butoxy)-4*H*-chromen-4-one (**IVe**).

### 3.2. In Vitro Cholinesterase Inhibitory Assay

The inhibitory activities of title compounds against commercially available cholinesterases (*ee*AChE) (Electrophorus electricus, Type V-S), *eq*BuChE (equine serum), were conducted using minor modifications of Ellman’s assay [29,32]. Nine different concentrations (1.2, 2.4, 4.7, 9.4, 19.8, 37.5, 75, 150, and 300 μM in H_2_O:DMSO (50:50)) of each test compound were used to determine the enzyme-inhibition activity. Briefly, 55 μL of 50 mM Tris-HCl buffer at pH 8, 25 μL of *eq*BuChE (0.03 U/mL), 20 μL of the test or standard compounds, and 125 μL of DTNB (0.8 mM) were incubated in 96-well plates at 37 °C for 15 min. Further, 25 μL of the substrate, BTCI (0.8 mM), was added, and the solution was incubated for additional 15 min. Finally, the absorbance was measured at 412 nm wavelength using a Microplate Spectrophotometry (TriStar^®^ S LB 942 model instrument). The IC_50_ values were determined from the absorbance obtained for various concentrations of the test and the standard compounds. All determinations were performed in triplicate.

To determine the % of inhibition ([I] = 100 μM), the substrate concentration (acetylthiocholine iodide for AChE; *S*-butyrylthiocholine iodide for BuChE) was fixed at 3 mM.

Lineweaver–Burk plot (1/V vs. 1/[BTCI]) was used for determining the inhibition constants (Ki’s) and the mechanism of action.

### 3.3. DPPH Antioxidant Assay

The colorimetric DPPH assay was carried out as described in [33]. The selected test compounds that showed promising *eq*BuChE inhibition and protective effects against H_2_O_2_ were selected for the DPPH assay, **IIf**, **IVa–e**, quercetin, and ascorbic acid. In brief, 30 μL of a test compound (in MeOH) was mixed with 200 μL of DPPH (100 μM in methanol) in a 96-well plate in triplicate. After 30 min incubation at room temperature in the absence of light, the absorbances were measured at 517 nm using Microplate Spectrophotometry (TriStar^®^ S LB 942 model instrument). The free radical scavenging activity results are expressed as a percentage of DPPH inhibition according to the following formula:

% of Inhibition = 100[(A(Blank) − A(sample))/A(blank)], where A(blank) consists of methanol (30 μL) mixed with DPPH (200 μL) absorbance, and A(sample) is the absorbance value for the added sample concentration compound mixed with DPPH. Ascorbic acid was used as a positive control.

### 3.4. Cytotoxicity Evaluation and Protective Assay against Hydrogen Peroxide-Induced Oxidative Stress in a Cellular Model

MCF-7 (Human Caucasian breast adenocarcinoma (MERCK—# 86012803-DNA-5UG), Database Name: ECACC collection, Accession Numbers: 86012803) was the cell line elected as a cell injury model for the study of the potential protective effect of the butyrylcholinesterase inhibitors (hybrids) against hydrogen peroxide-induced oxidative stress. Cytotoxicity of the hybrids was also evaluated.

#### 3.4.1. Preparation of the Solutions with the Compounds

The compounds to be tested were firstly dissolved in dimethyl sulfoxide (DMSO) at a concentration of 4 × 10^−2^ M. They were then diluted in cell growth media (EMEM) (supplemented with FBS 10%) with 0.25% DMSO to obtain solutions between 6.25 and 50.00 µM.

#### 3.4.2. Cell Culture

Eagle’s Minimum Essentia Medium (EMEM), including 10% fetal bovine serum (FBS), 100 IU/mL penicillin, and 100 μg/mL streptomycin, was used to culture MCF-7 cells at 37 °C in 5% CO_2_ atmosphere incubator with media replenished every 2 days. A total of 5 × 10^3^ cells were cultured in 96-well plates and incubated for 72 h prior to stimulus application.

#### 3.4.3. Stimulus Application and Cellular Viability Assessment

The culture medium was subsequently replaced with different final concentrations (6.25–50 μM) of the compounds to be tested. Negative controls were prepared, incubating cells in culture media. Galantamine and quercetin in equivalent concentration range were also applied as positive controls.

Parallel experiments were performed for cytotoxicity assessment and for protective effect against hydrogen peroxide-induced oxidative stress evaluation. First, 10 μL of PBS was added in the designated wells; second, 10 μL of H_2_O_2_ (reaching a final concentration of 100 µM) was added. Following a 2 h incubation period at 37 °C in 5% CO_2_ atmosphere incubator, cellular viability was measured by adding WST-1 dehydrogenases substrate and incubating for 1 h, and the amount of formazan dye produced was evaluated by measuring the absorbance at 440 nm.

### 3.5. Artemia Salina Lethal Toxicity Assay

The ARTOXKIT M protocol was used. The percentage of dyed nauplii of *Artemia salina*, grown in the presence of variable concentrations of the inhibitor compounds, was measured to determine the LC_50_ values. A test with K_2_Cr_2_O_7_ was used for quality control.

### 3.6. Statistical Analysis

All experimental results are shown as the mean ± SD. Comparisons between groups were performed using Tukey–Kramer Multiple Comparison Test performed in NCSS 2023 Statistical Software (2023) (NCSS, LLC. Kaysville, UT, USA, version 25, S Inc., Chicago, IL, USA), and statistical significance was considered as *p* < 0.05. Origin 9.0 software (OriginLab, Northampton, MA, USA) and Microsoft^®^ Excel^®^ (for Microsoft 365 MSO, version 2307 Build 16. 0. 16626. 20198, 64-bit) were used for drawing and data analysis in this paper.

## 4. Conclusions

The results show that some of these new quercetin-1,2,3-triazole hybrids (**II** and **IV**) have better anti-BuChE activity than galantamine itself. The best inhibitors of *eq*BuChE are hybrids **IIe, IIf, IVa** and **IVb,** having displayed IC_50_ values of 26.6, 11.2, 24.7, and 32.4 μM, respectively, and are more potent inhibitors than galantamine (38 μM). Comparing the structures of hybrids **IIe** and **IIf** with **IVa** and **IVb**, the former have the hydroxyl groups of quercetin methylated, but at the 4-position of the 1,2,3-triazole ring, they have thymol (**IIe**) and isatin (**IIf**) units, which are recognized as important pharmacophores in medicinal chemistry. Although both are aromatic, isatin bears two carbonyl groups, which are probably the location for binding to the amino acid residues of the enzyme’s catalytic active site. In the case of hybrids **IVa** and **IVb**, quercetin has free hydroxyl groups and, with the phenyl (**IVa**) and cyclopropyl (**IVb**) substituents in the 4-position of the 1,2,3-triazole, the free hydroxyl groups are beneficial for binding to the enzyme’s active site, but the flat planar phenyl group allows for a better fit than the more three-dimensionally shaped cyclopropyl group, with possibly more steric hindrance.

Indeed, the main difference between **IVb** and **IVd** (competitive and non-competitive inhibition mechanism, respectively) lies in the substituents at the 4-position of the 1,2,3-triazole ring, the small but compact cyclopropyl that favors the binding of hybrid **IVb** outside the active center, leading to a mixed type (non-competitive) enzymatic inhibition mechanism for this compound.

Hybrid **IIf** is a better BuChE inhibitor than quercetin, but hybrids **IIe**, **IVa,** and **IVb** are slightly weaker than quercetin. It seems that the presence of the isatin group in the structure of the hybrid **IIf** is the key to improving the quercetin biologic activity.

Hybrids **IVa, b, d, e** have potent antioxidant activity in vitro, like quercetin, at a concentration of 100 μg/mL. Both hybrids have free quercetin hydroxyls, indicating that they are the main scavengers of the DPPH free radical.

When studied in a cellular model, quercetin showed low capacity in cellular protection against hydrogen peroxide-induced oxidative stress, which is probably the result of poor cellular permeability. The quercetin hybrids seem to be able to overcome this difficulty, and compounds **IIf**, **IVb,** and **IVd** were effective in protecting cells against hydrogen peroxide-induced oxidative stress. Furthermore, compound **IIf,** although ineffective in radical scavenging, also presents a good cellular antioxidant-protection capacity, probably associated with its isatin unit.

The good cellular anti-oxidative protection showed by compounds **IIf**, **IVb,** and **IVd**, combined with low cytotoxicity and low general toxicity in *Artemia salina*, underline the interest of these compounds as multitarget drugs.

To summarize, hybrids **IIf**, **IVb,** and **IVd** showed potent inhibitory activity, low toxicity, antioxidant, and protective antioxidant effects and did not affect cell viability, making them a promising contribution to the treatment of Alzheimer’s disease. Undoubtedly, these new quercetin-1,2,3-trizole hybrids are also promising multifunctional agents.

## Figures and Tables

**Table 1:** Screening of the quercetin-1,2,3-triazole hybrids **I–IV** against *eq*BuChE and *ee*AChE at a concentration of 100 µM.

Compound	% Inhibition	
	*ee*AChE	*eq*BuChE
**Ia**	0.00	54.39
**Ib**	0.00	63.33
**IIa**	7.75	55.26
**IIb**	0.00	54.42
**IIc**	0.00	69.59
**IId**	0.00	60.59
**IIe**	4.13	81.70
**IIf**	24.31	100.00
**IIg**	0.00	53.84
**IIh**	38.57	7.97
**IIi**	0.00	74.40
**IIIa**	40.57	46.85
**IIIc**	17.19	26.86
**IIId**	15.07	27.32
**IIIf**	0.00	34.51
**IVa**	0.00	82.81
**IVb**	5.21	90.46
**IVc**	21.55	69.92
**IVd**	3.65	56.58
**IVe**	29.05	63.41
Quercetin	8.18	100.00

**Table 2:** IC_50_ values for *eq*BuChE with quercetin-1,2,3-triazole hybrids **II** and **IV**.

Compound	IC_50_ Values (µM)
**IIa**	>300
**IIb**	>300
**IIc**	>300
**IId**	>300
**IIe**	26.6
**IIf**	11.2
**IIg**	>300
**IIh**	>300
**IIi**	70.20
**IVa**	24.7
**IVb**	32.4
**IVc**	65.7
**IVd**	39.0
**IVe**	>150
Galantamine	37.6
Quercetin	18.2

**Table 3:** Kinetic parameters and inhibitory constant, Ki, for hybrids **IIf**, **IVb,** and **IVd**.

		K_M_ or K_M_ ^app^ * (mM)	V_max_ or V_max_ ^app^ * (U.mg^−1^)	Inhibition Type and K_i_ *
Without inhibitor		0.4 ± 0.1	11.9 ± 2.3	-
**IIf**	5 µM	1.5	11.8	Competitive K_i_ = 6.2 µM
	20 µM	2.6	11.4	
	40 µM	3.9	13.4	
**IVb**	17.5 µM	0.57	7.5	Mixed (non-competitive) K_i_ = 30 µM
	35 µM	0.53	6.5	
	70 µM	0.52	4.5	
**IVd**	17.5 µM	1.0	9.6	Competitive K_i_ = 36 µM
	35 µM	1.6	9.0	

* K_M_—Michaelis constant; K_M_ ^app^—apparent Michaelis constant; V_max_—maximal velocity; V_max_ ^app^—apparent maximal velocity; K_i_—inhibitory constant.

**Table 4:** Lethal concentration at 50% (LC_50_) of tested compounds.

Compound	LC_50_
**K_2_Cr_2_O_7_**	22.7 mg/L
**IVb**	10–100 µM
**IVd**	≥100 µM
**IIf**	34.6 µM

## Data Availability

Data are contained within the article and supplementary materials.

## Supplementary Materials

The following supporting information can be downloaded at: https://www.mdpi.com/article/10.3390/molecules28227495/s1.
