# Supplementary material for: Quercetin-1,2,3-Triazole Hybrids as Multifunctional Anti-Alzheimer’s Agents
Source: Molecules. 2023 Nov 9;28(22):7495. doi: 10.3390/molecules28227495 (PMC10673615; doi:10.3390/molecules28227495)

Supporting Information

Quercetin-1,2,3-Triazole Hybrids as Multifunctional Anti-Alzheimer's Agents

Elisabete P. Carreiro [1,*], Ana R. Costa [2,3,4,*], Célia M. Antunes [2,3,4], Sofia Ernesto [5], Flávia Pinto [5], Beatriz Rodrigues [5] and Anthony J. Burke [5,6]

1 Institute for Research and Advanced Training (IIFA), LAQV-REQUIMTE, University of Évora, Rua Romão Ramalho, 59, 7000-671 Évora, Portugal
2 Department of Medical and Health Sciences, School of Health and Human Development, University of Évora, Rua Romão Ramalho 59, 7000-671 Évora, Portugal; cmma@uevora.pt
3. Institute of Earth Sciences, Institute of Research and Advanced Training, University of Évora, 7000-671 Évora, Portugal
4 Academic Clinical Center of Alentejo, C-TRAIL, Rua Romão Ramalho, 59, 7000-671 Évora, Portugal
5 Department of Chemistry and Biochemistry, School of Sciences and Technologies, University of Évora, Rua Romão Ramalho 59, 7000-671 Évora, Portugal; sfcernesto@gmail.com (S.E.); flaviaisabelmp@gmail.com (F.P.); l48782@alunos.uevora.pt (B.R.); ajburke@ff.uc.pt (A.J.B.)
6 Faculty Pharmacy, University of Coimbra, Pólo das Ciências da Saúde, Azinhaga de Santa Comba, 3000-548 Coimbra, Portugal
* Correspondence: betepc@uevora.pt (E.P.C.); acrc@uevora.pt (A.R.C.)

Contents: **pages**

**1. ^{1}H NMR spectra of hybrids quercetin-1,2,3-triazole I-IV; General Remarks**	**1**
**1.1. Hybrids Ia-b**	**2**
**1.2. Hybrids IIa-i**	**3 - 7**
**1.3. Hybrids IIIa-g**	**7 - 10**
**1.4. Hybrids IVa-e**	**11 -13**

1. ^{1}H NMR spectra of hybrids quercetin-1,2,3-triazole: General Remarks

The 23 quercetin-1,2,3-triazole **I-IV** hybrids, the target molecules of this work, were characterized by ^{1}H NMR to verify their structures. ^{1}H NMR spectra were recorded with a Bruker Avance III at 400 MHz. Chemical shifts were quoted in parts per million (ppm) and referenced to the appropriate solvent peak.

1.1. Hybrids Ia-b

1.1.1. 3-((1-Benzyl-1H-1,2,3-triazol-4-yl)methoxy)-2-(3,4-dimethoxyphenyl)-5,7-dimethoxy-4H-chromen-4-one Ia; ^{1}H NMR (400 MHz, $CDCl_3$):

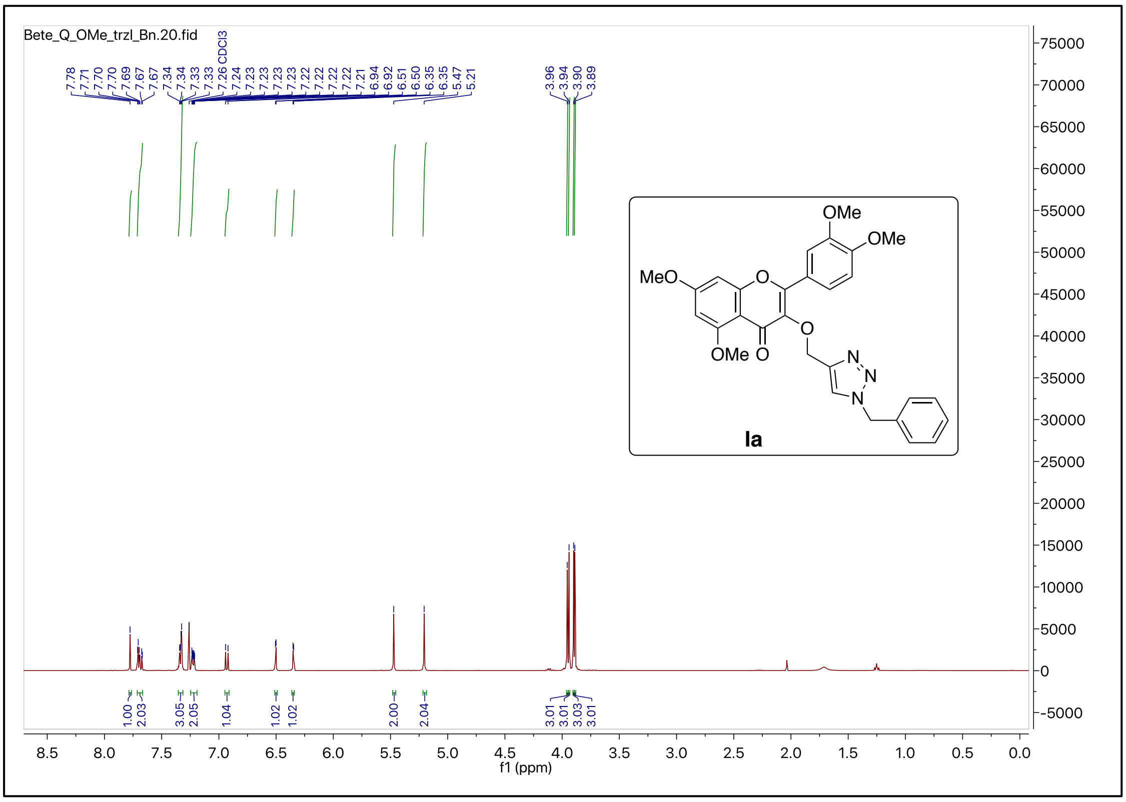

1.1.2. 3-((1-(3-Chlorobenzyl)-1H-1,2,3-triazol-4-yl)methoxy)-2-(3,4-dimethoxyphenyl)-5,7-dimethoxy-4H-chromen-4-one Ib; ^{1}H NMR (400 MHz, $CDCl_3$):

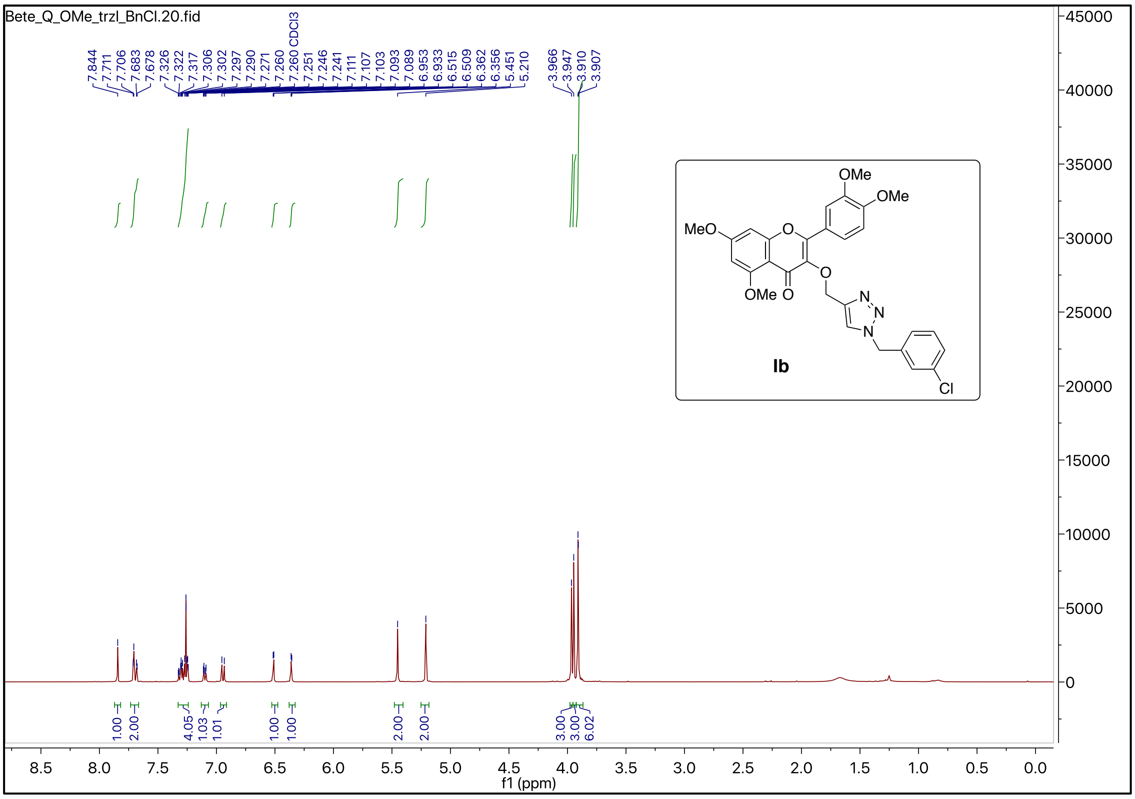

1.2. Hybrids II

1.2.1. 2-(3,4-Dimethoxyphenyl)-5,7-dimethoxy-3-(4-(4-phenyl-1H-1,2,3-triazol-1-yl)butoxy)-4H-chromen-4-one IIa, ^{1}H NMR (400 MHz, DMSO-d$_6$):

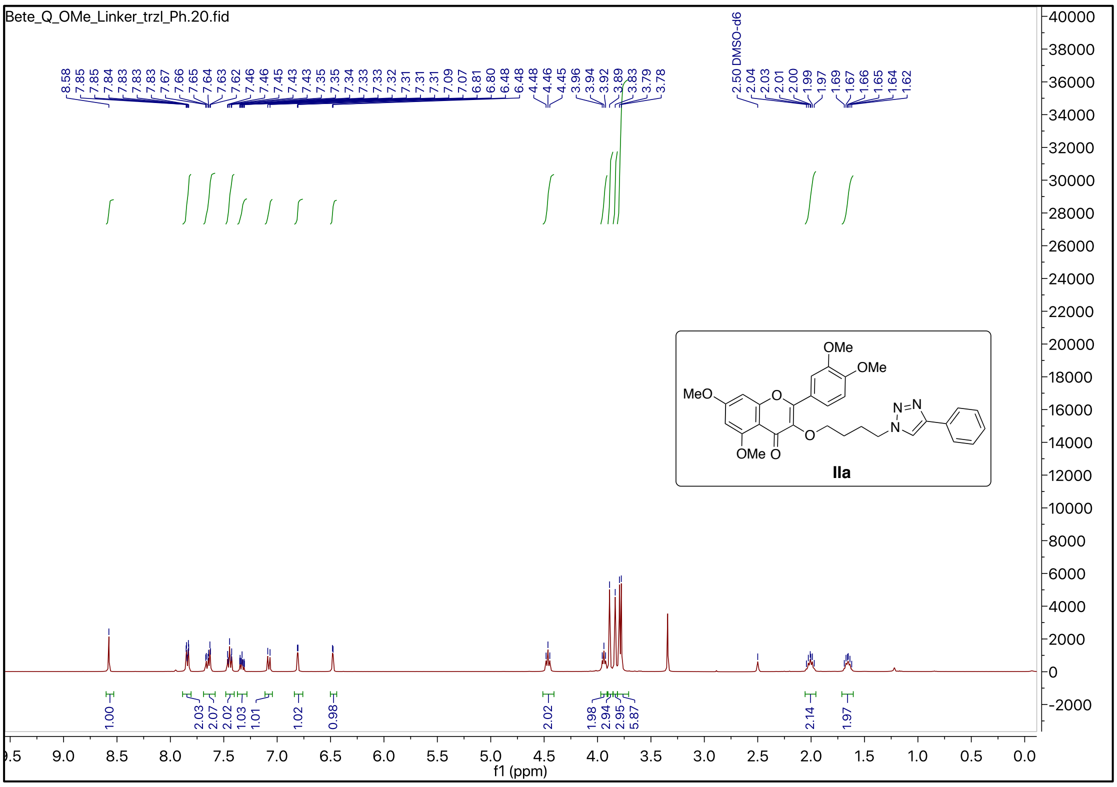

1.2.2. 2-(3,4-Dimethoxyphenyl)-3-(4-(4-cyclopropyl-1H-1,2,3-triazol-1-yl)butoxy)-5,7-dimethoxy-4H-chromen-4-one IIb; ^{1}H NMR (400 MHz, DMSO-d$_6$):

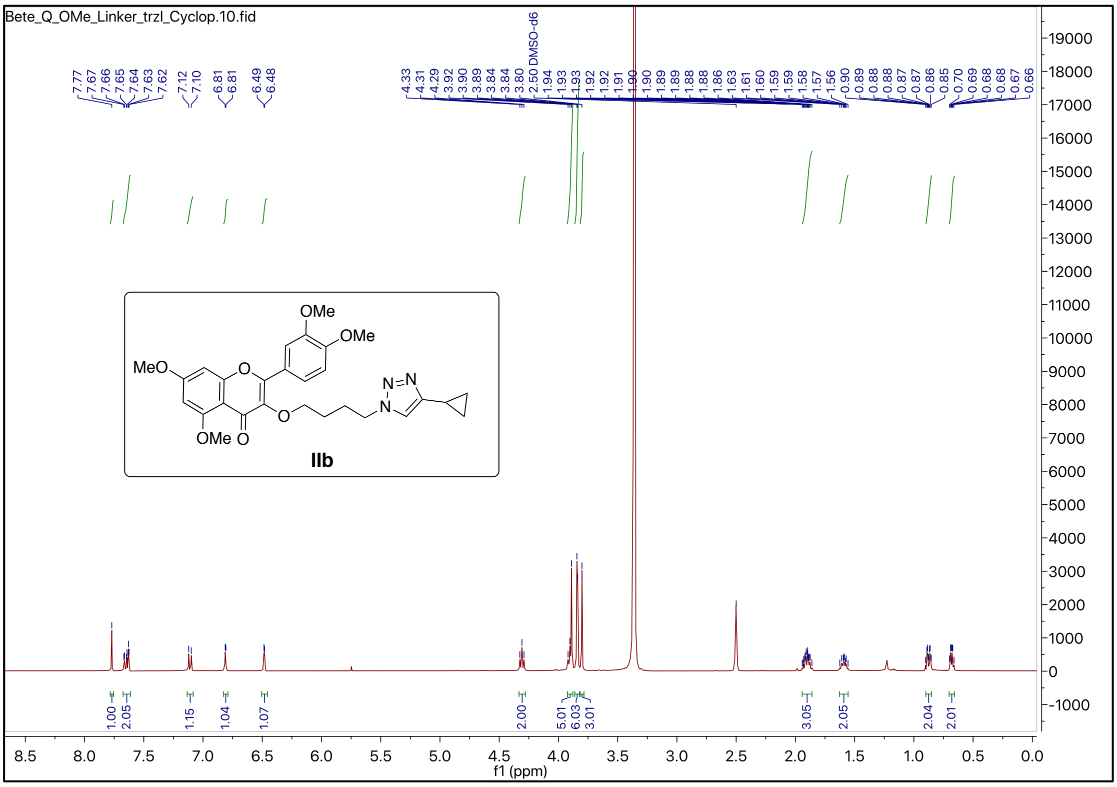

1.2.3. 2-(3,4-Dimethoxyphenyl)-3-(4-(4-(1-hydroxycyclopentyl)-1H-1,2,3-triazol-1-yl)butoxy)-5,7-dimethoxy-4H-chromen-4-one IIc; ^{1}H NMR (400 MHz, DMSO-d_6):

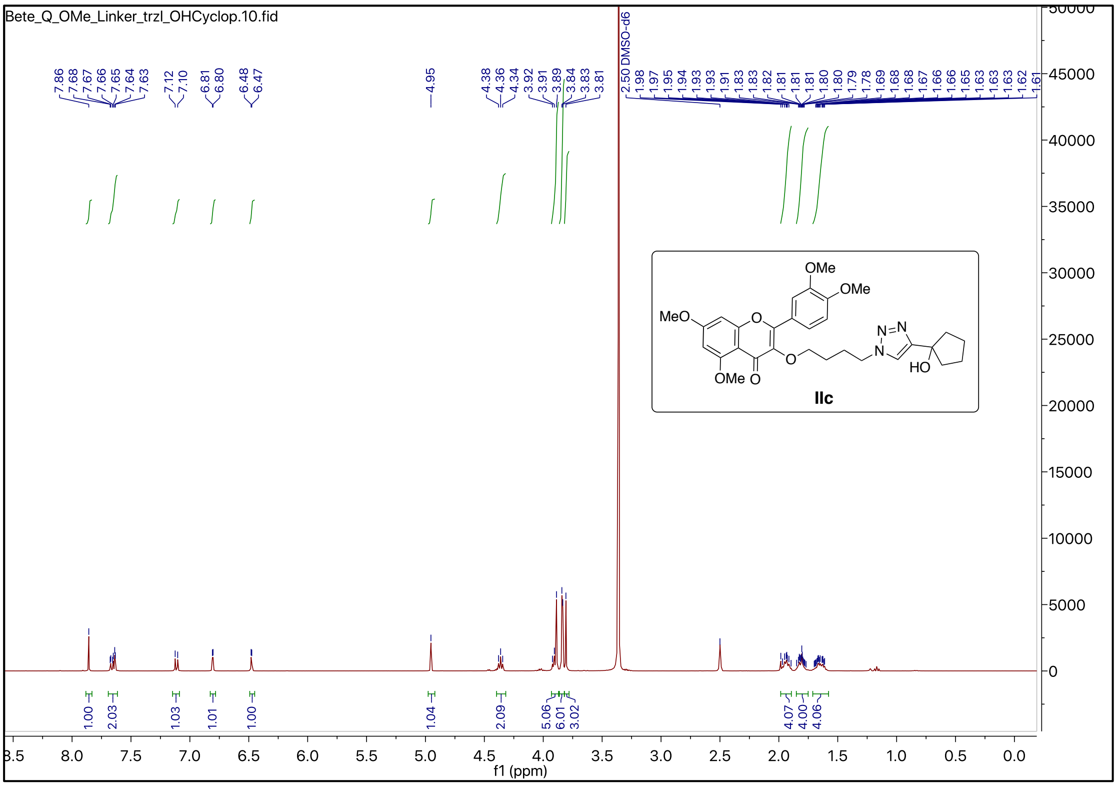

1.2.4. 2-(3,4-Dimethoxyphenyl)-3-(4-(4-(2-hydroxypropan-2-yl)-1H-1,2,3-triazol-1-yl)butoxy)-5,7-dimethoxy-4H-chromen-4-one IId; ^{1}H NMR (400 MHz, DMSO-d_6):

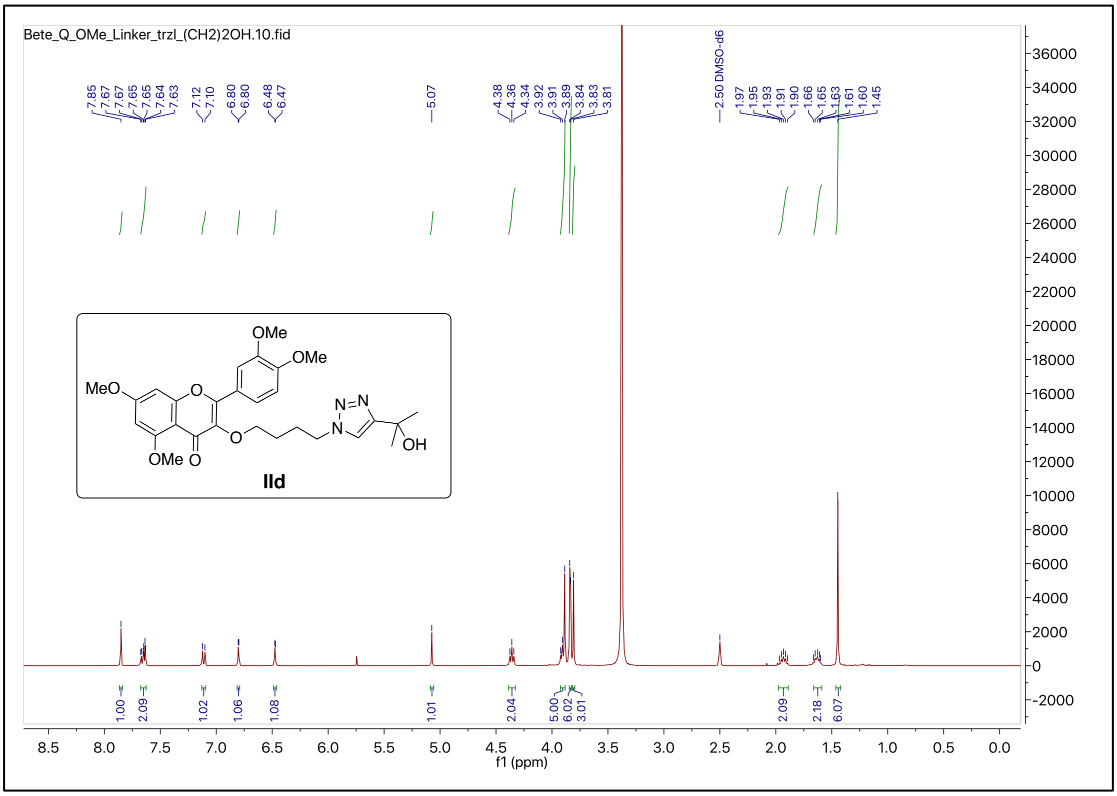

1.2.5. 2-(3,4-Dimethoxyphenyl)-3-(4-(4-((2-isopropyl-5-methylphenoxy)methyl)-1H-1,2,3-triazol-1-yl)butoxy)-5,7-dimethoxy-4H-chromen-4-one IIe; ^{1}H NMR (400 MHz, $CDCl_3$):

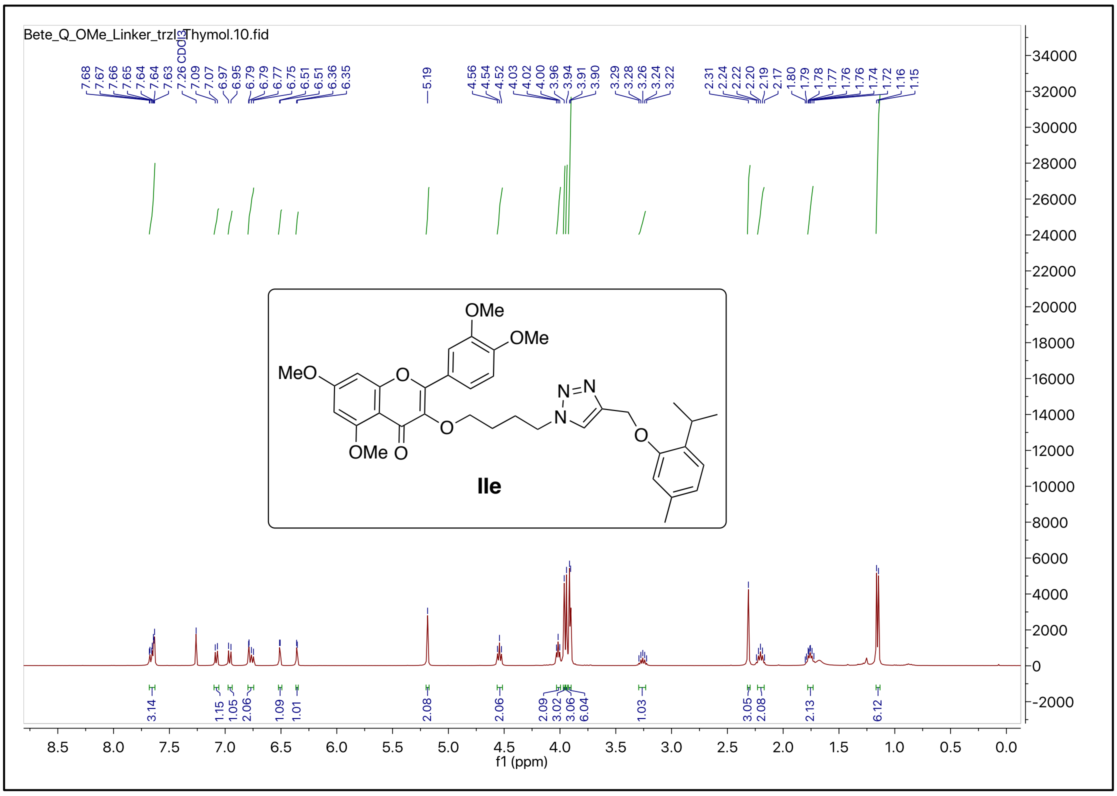

1.2.6. 1-((1-(4-((2-(3,4-Dimethoxyphenyl)-5,7-dimethoxy-4-oxo-4H-chromen-3-yl)oxy)butyl)-1H-1,2,3-triazol-4-yl)methyl)indoline-2,3-dione IIf; ^{1}H NMR (400 MHz, DMSO-d_6):

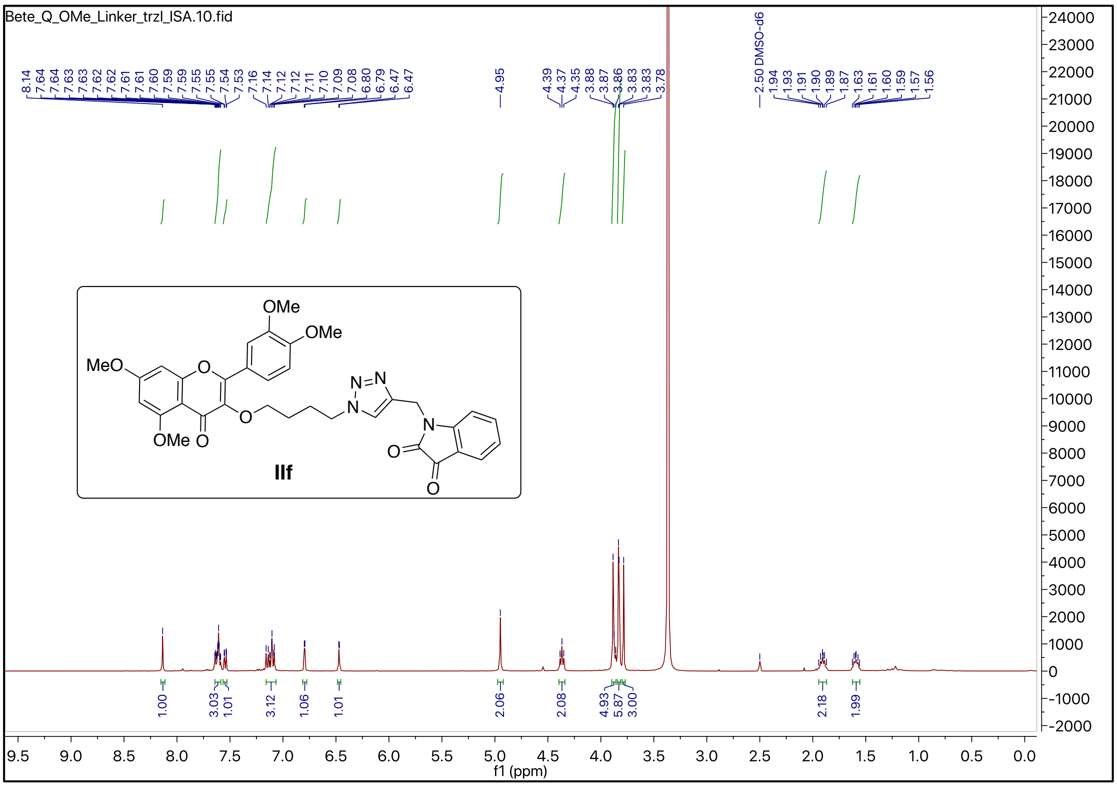

1.2.7. 2-(3,4-Dimethoxyphenyl)-3-(4-(4-(hydroxymethyl)-1H-1,2,3-triazol-1-yl)butoxy)-5,7-dimethoxy-4H-chromen-4-one IIg: ^{1}H NMR (400 MHz, DMSO-d_6):

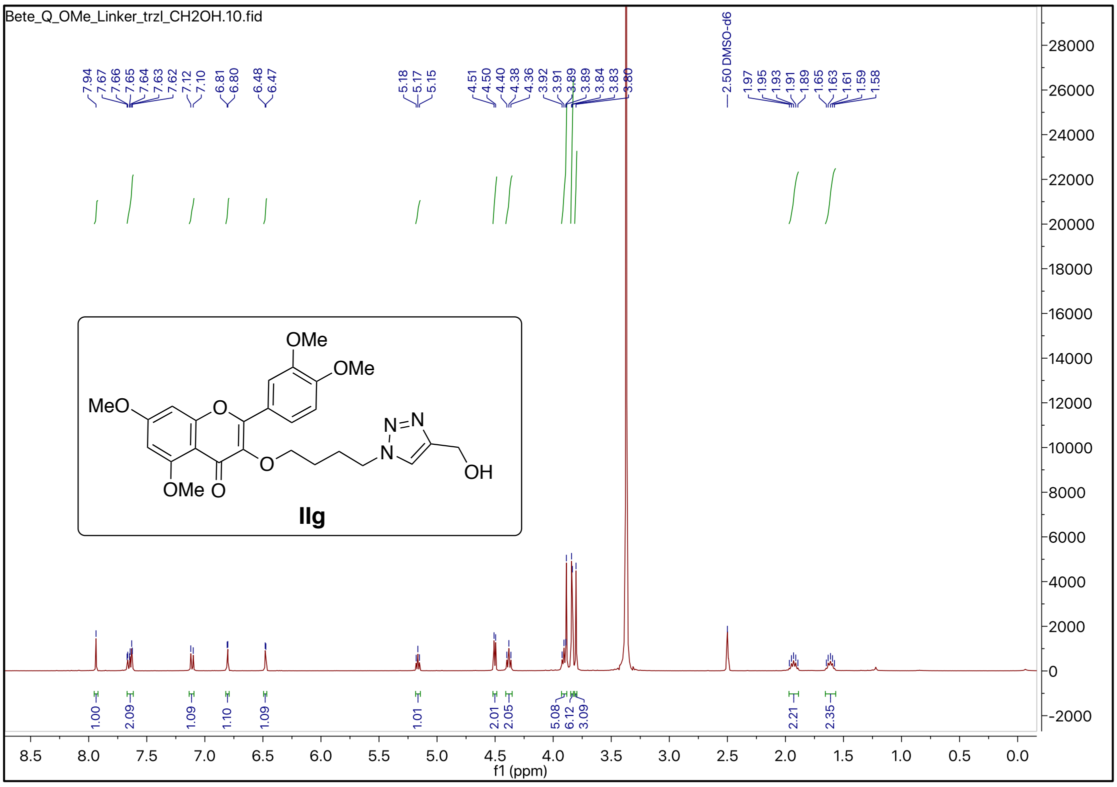

1.2.8. 2-(3,4-Dimethoxyphenyl)-5,7-dimethoxy-3-(4-(4-propyl-1H-1,2,3-triazol-1-yl)butoxy)-4H-chromen-4-one IIh; ^{1}H NMR (400 MHz, $CDCl_3$):

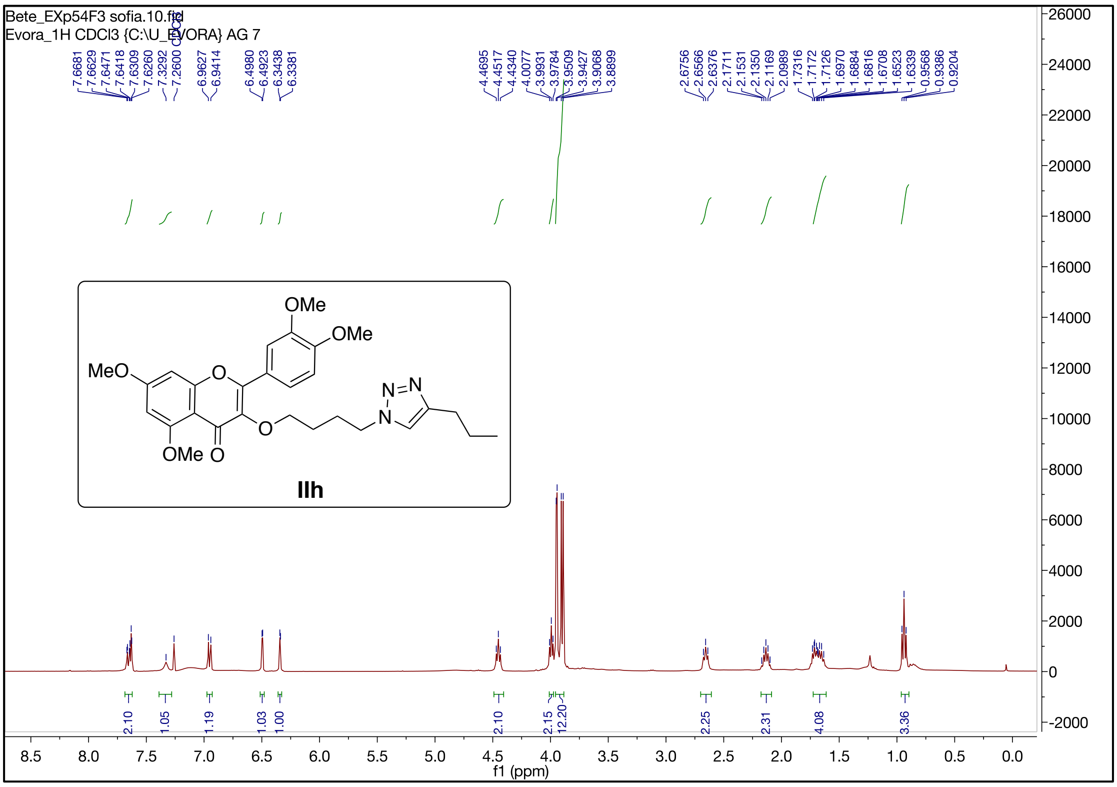

1.2.9. 3-(4-(4-(2-Aminopropan-2-yl)-1H-1,2,3-triazol-1-yl)butoxy)-2-(3,4-dimethoxyphenyl)-5,7-dimethoxy-4H-chromen-4-one IIi; ^{1}H NMR (400 MHz, DMSO-d_6):

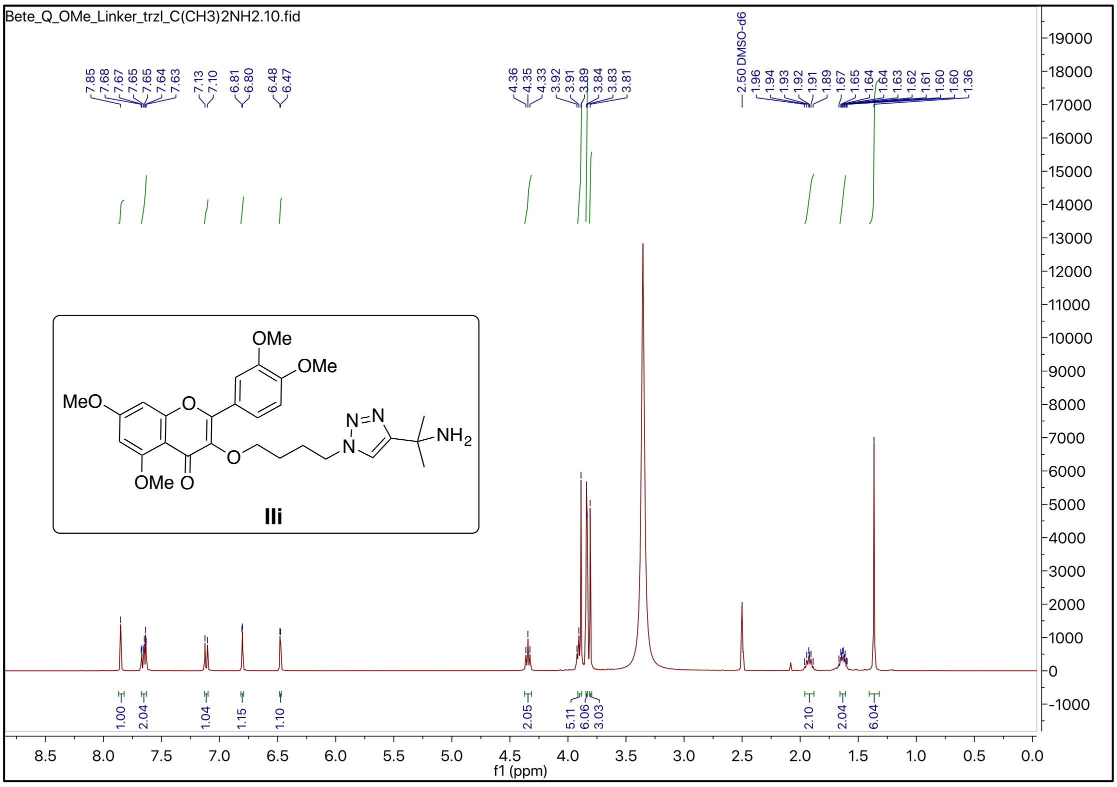

1.3. Hybrids III

1.3.1. 5,7-Bis(benzyloxy)-2-(3,4-bis(benzyloxy)phenyl)-3-(4-(4-phenyl-1H-1,2,3-triazol-1-yl)butoxy)-4H-chromen-4-one IIIa; ^{1}H NMR (400 MHz, CDCl$_3$):

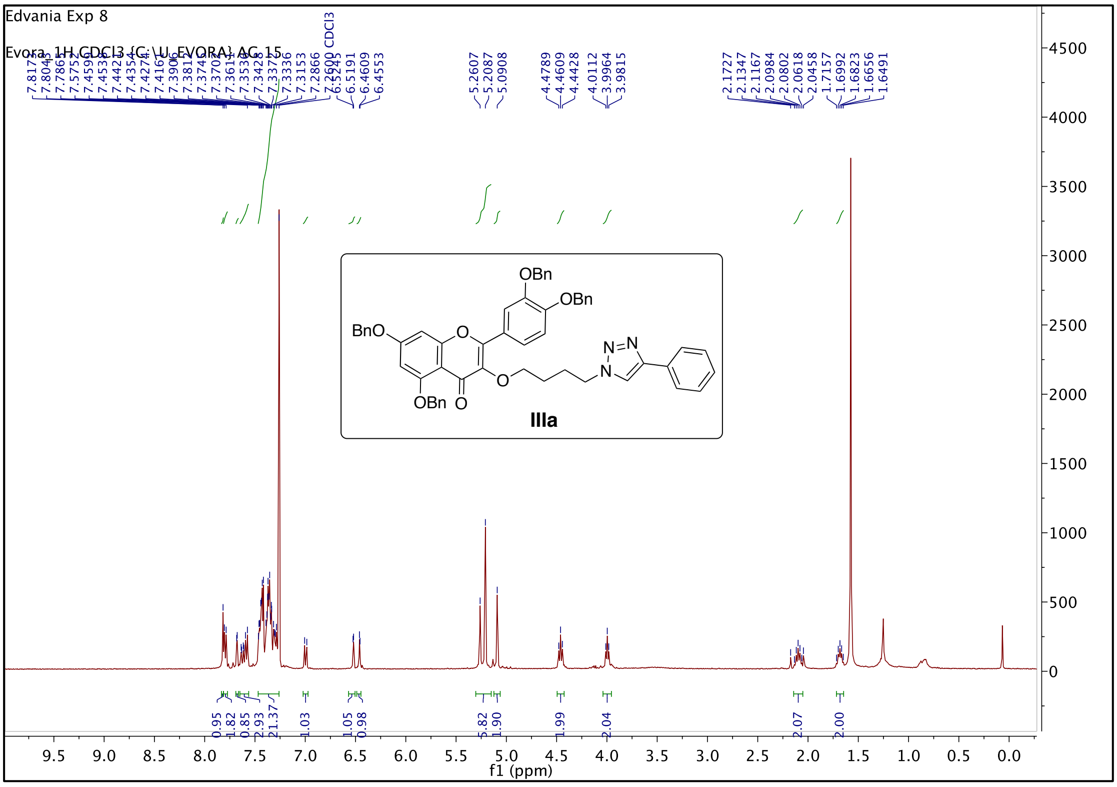

1.3.2. 5,7-Bis(benzyloxy)-2-(3,4-bis(benzyloxy)phenyl)-3-(4-(4-cyclopropyl-1H-1,2,3-triazol-1-yl)butoxy)-4H-chromen-4-one IIIb; ^{1}H NMR (400 MHz, $CDCl_3$):

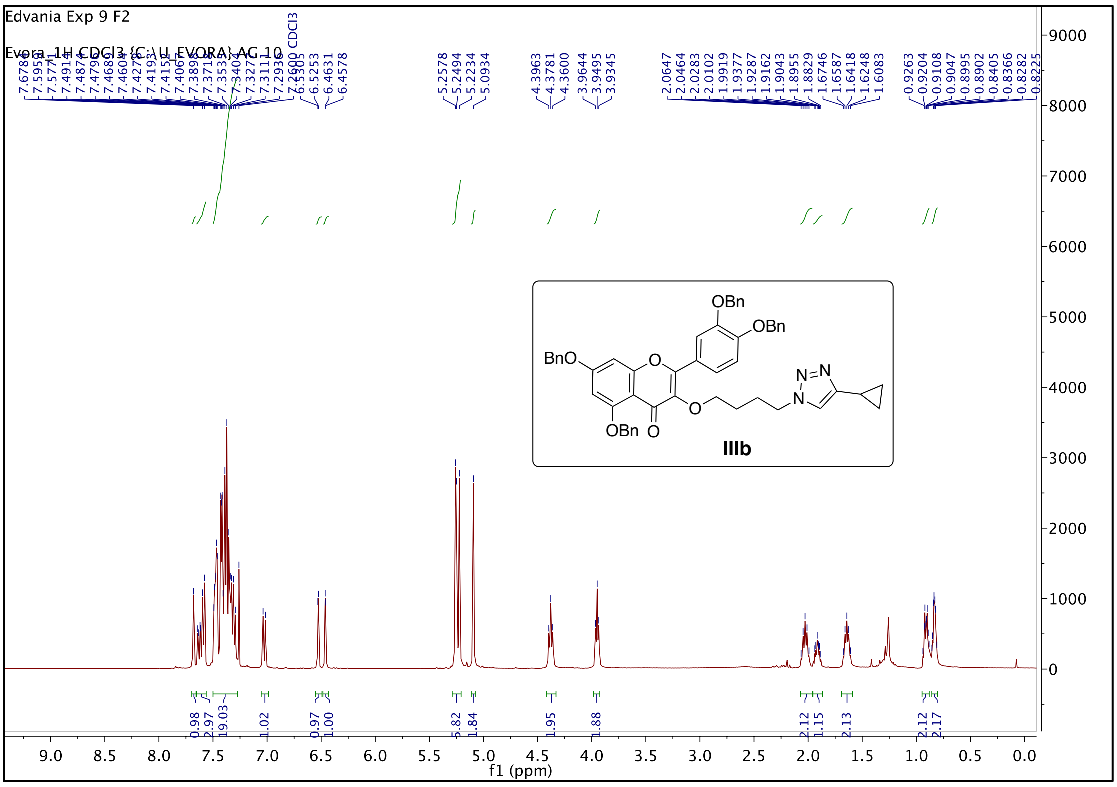

1.3.3. 5,7-Bis(benzyloxy)-2-(3,4-bis(benzyloxy)phenyl)-3-(4-(4-(1-hydroxycyclopentyl)-1H-1,2,3-triazol-1-yl)butoxy)-4H-chromen-4-one IIIc; ^{1}H NMR (400 MHz, DMSO-d_6):

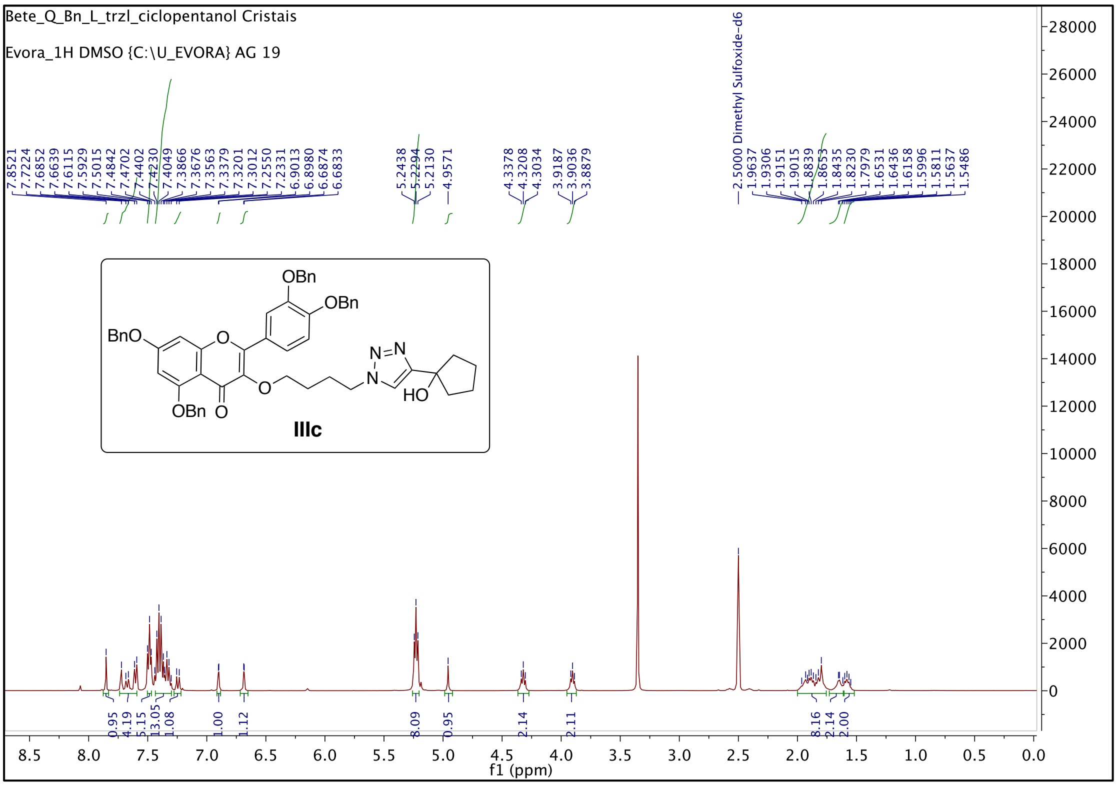

1.3.4. 5,7-Bis(benzyloxy)-2-(3,4-bis(benzyloxy)phenyl)-3-(4-(4-(2-hydroxypropan-2-yl)-1H-1,2,3-triazol-1-yl)butoxy)-4H-chromen-4-one IIId; ^{1}H NMR (400 MHz, $CDCl_3$):

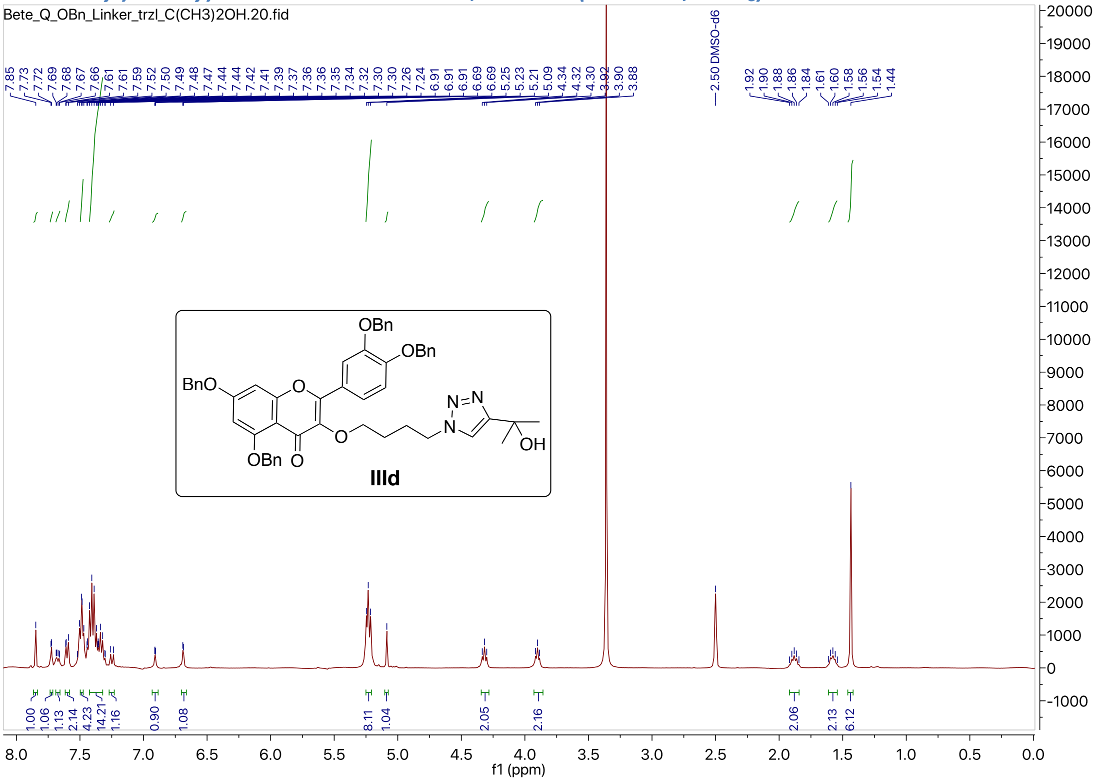

1.3.5. 5,7-Bis(benzyloxy)-2-(3,4-bis(benzyloxy)phenyl)-3-(4-(4-((2-isopropyl-5-methylphenoxy)methyl)-1H-1,2,3-triazol-1-yl)butoxy)-4H-chromen-4-one IIIe; ^{1}H NMR (400 MHz, $CDCl_3$):

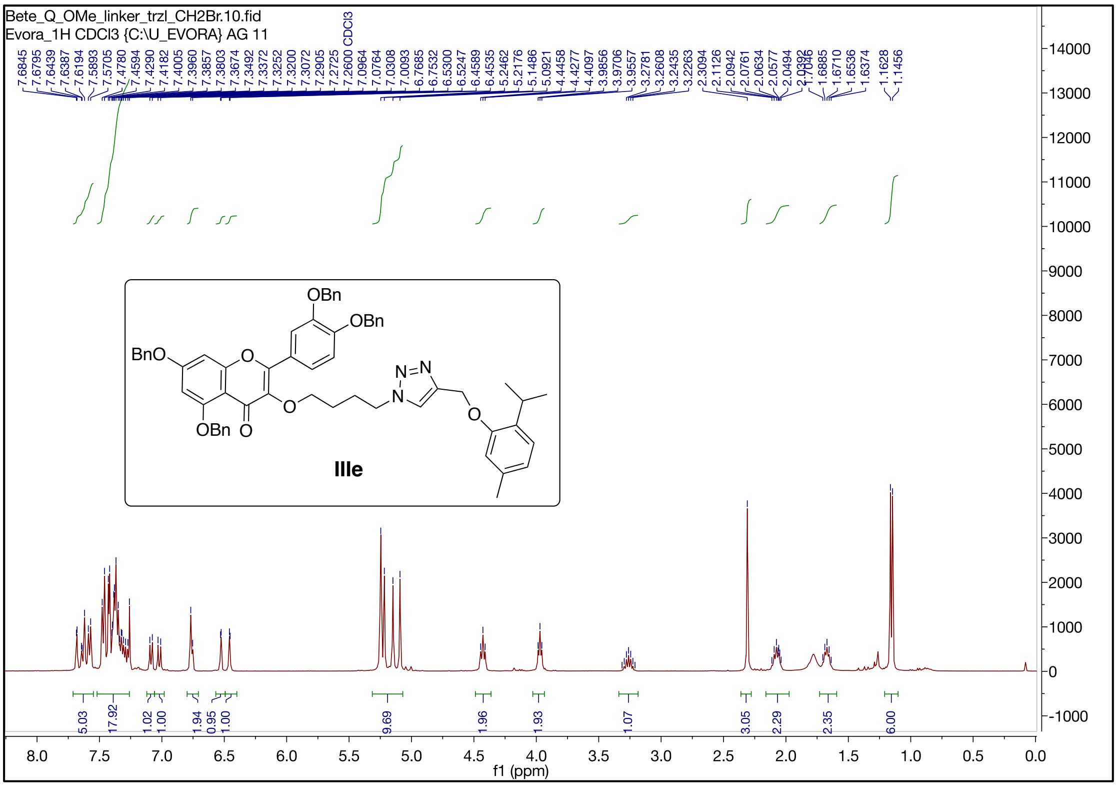

1.3.6. 1-((1-(4-((5,7-Bis(benzyloxy)-2-(3,4-bis(benzyloxy)phenyl)-4-oxo-4H-chromen-3-yl)oxy)butyl)-1H-1,2,3-triazol-4-yl)methyl)indoline-2,3-dione IIIf; ^{1}H NMR (400 MHz, DMSO-d_6):

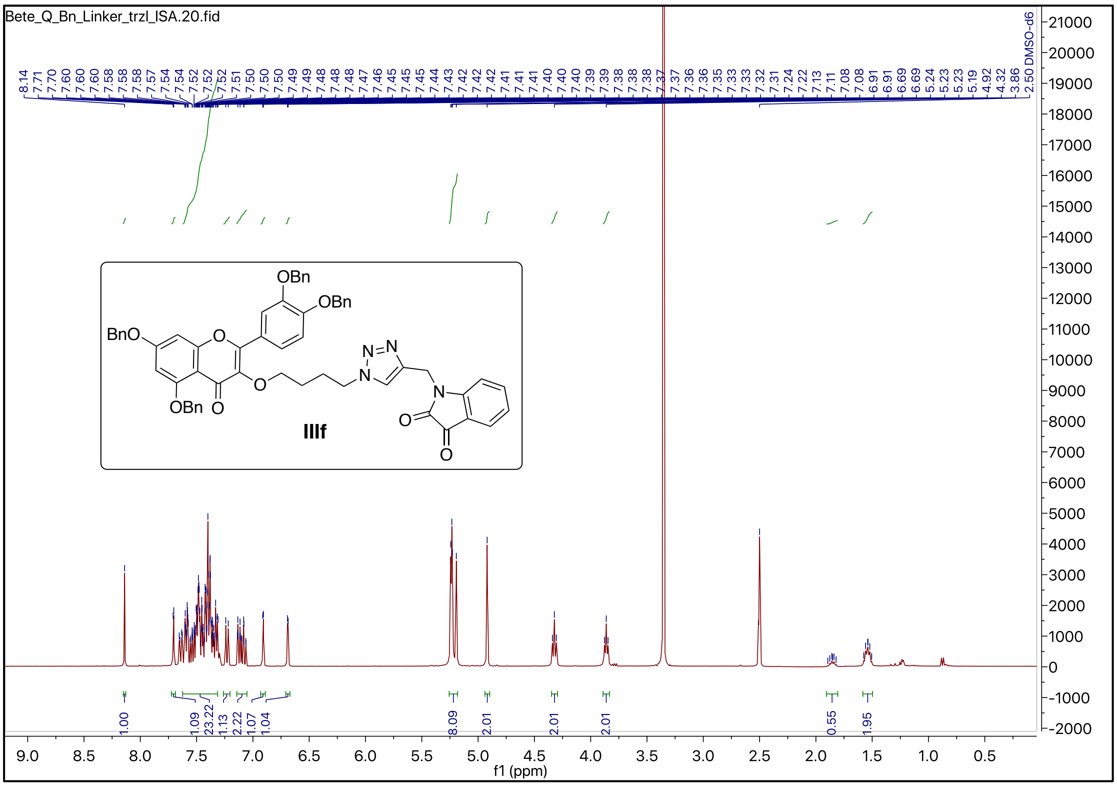

1.3.7. 5,7-Bis(benzyloxy)-2-(3,4-bis(benzyloxy)phenyl)-3-(4-(4-(hydroxymethyl)-1H-1,2,3-triazol-1-yl)butoxy)-4H-chromen-4-one IIIg; ^{1}H NMR (400 MHz, DMSO-d_6):

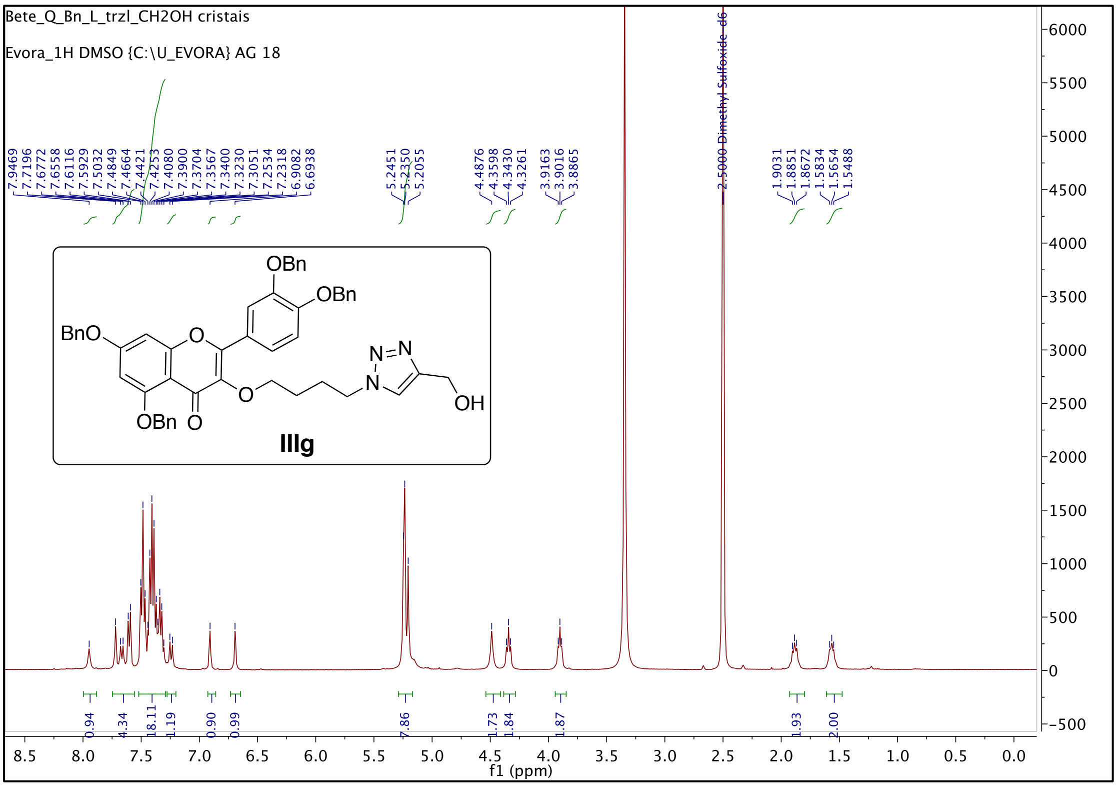

1.4. Hybrids IV

1.4.1. 2-(3,4-Dihydroxyphenyl)-5,7-dihydroxy-3-(4-(4-phenyl-1H-1,2,3-triazol-1-yl)butoxy)-4H-chromen-4-one IVa; ^{1}H NMR (400 MHz, DMSO-d_6):

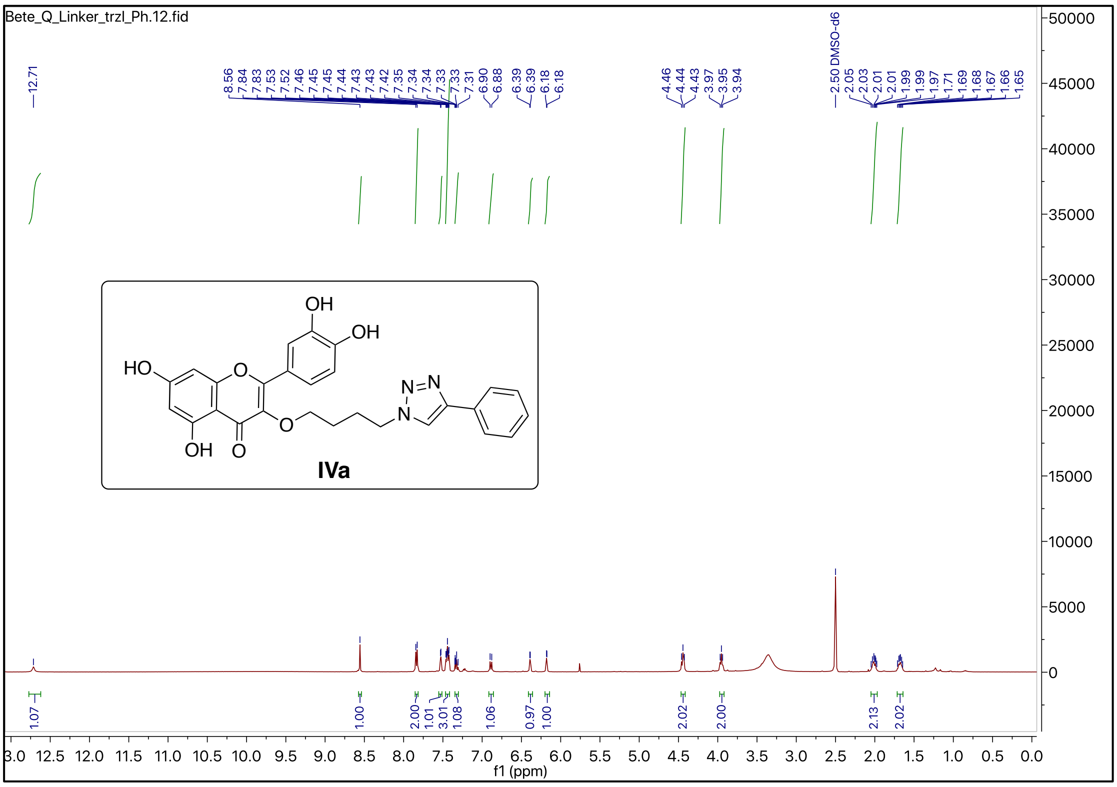

1.4.2. 3-(4-(4-Cyclopropyl-1H-1,2,3-triazol-1-yl)butoxy)-2-(3,4-dihydroxyphenyl)-5,7-dihydroxy-4H-chromen-4-one IVb; ^{1}H NMR (400 MHz, DMSO-d_6):

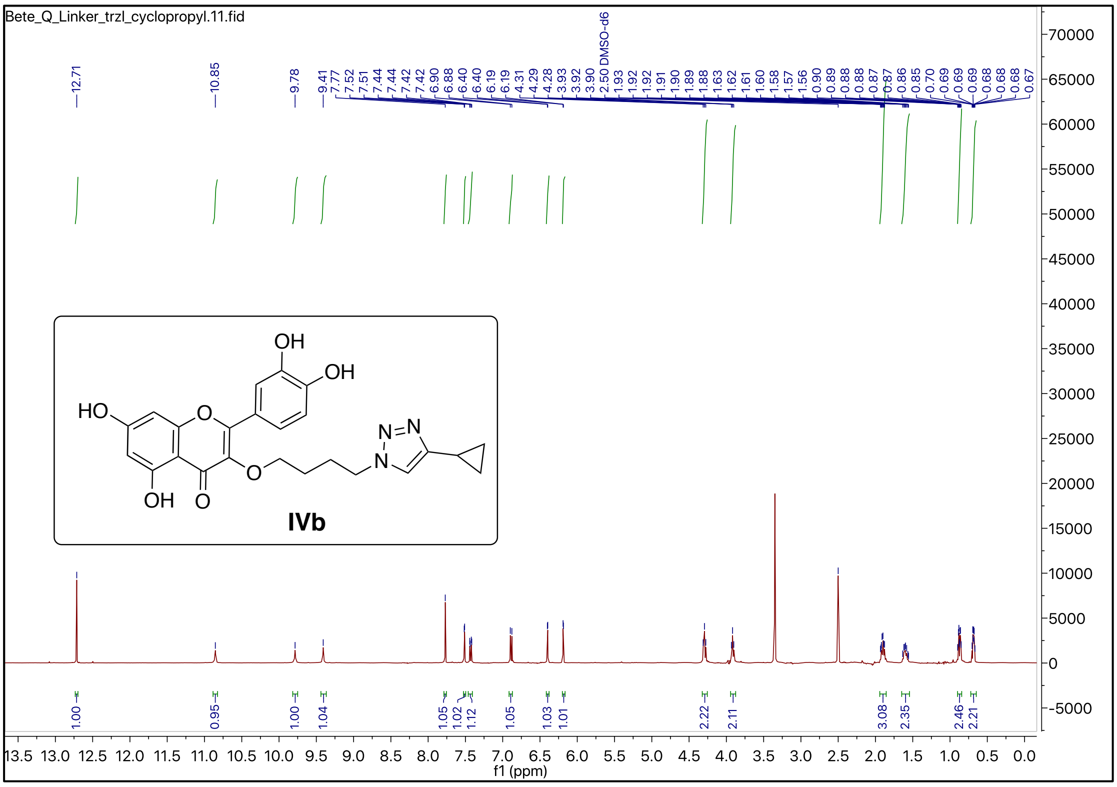

1.4.3. 2-(3,4-Dihydroxyphenyl)-5,7-dihydroxy-3-(4-(4-(1-hydroxycyclopentyl)-1H-1,2,3-triazol-1-yl)butoxy)-4H-chromen-4-one IVc; ^{1}H NMR (400 MHz, $(CD_3)_2CO$-d_6):

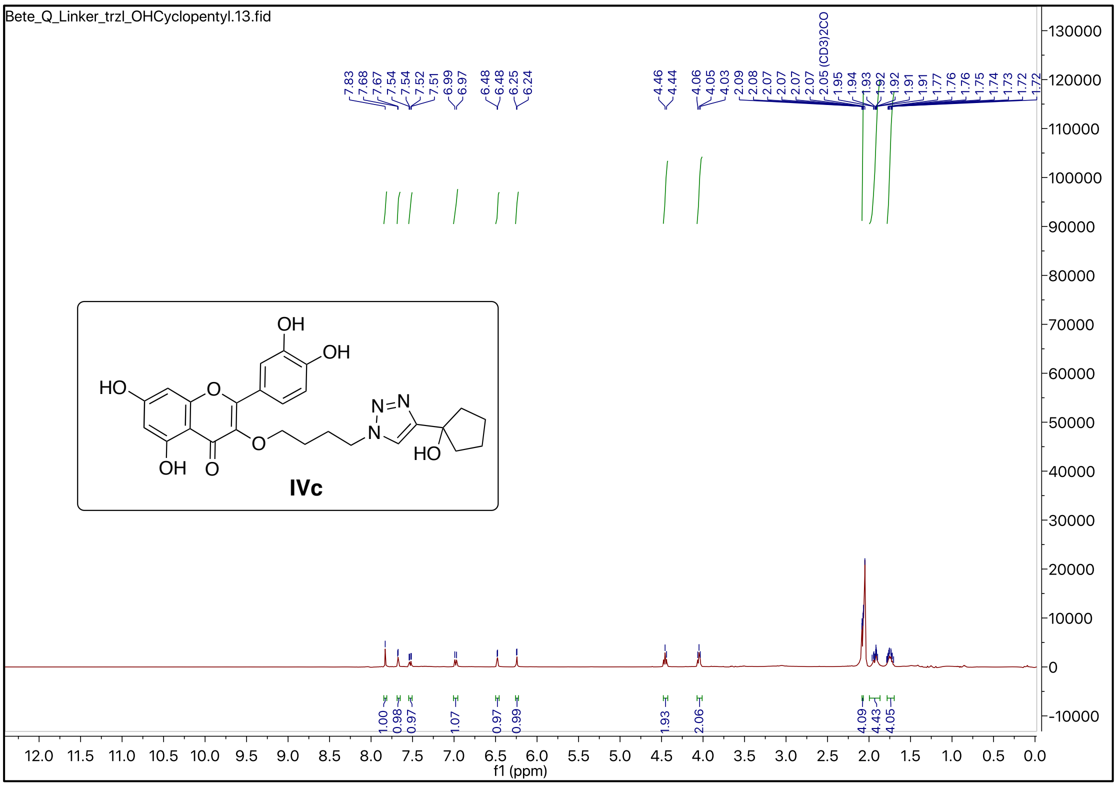

1.4.4. 2-(3,4-Dihydroxyphenyl)-5,7-dihydroxy-3-(4-(4-(2-hydroxypropan-2-yl)-1H-1,2,3-triazol-1-yl)butoxy)-4H-chromen-4-one IVd; ^{1}H NMR (400 MHz, $(CD_3)_2O$-d_6):

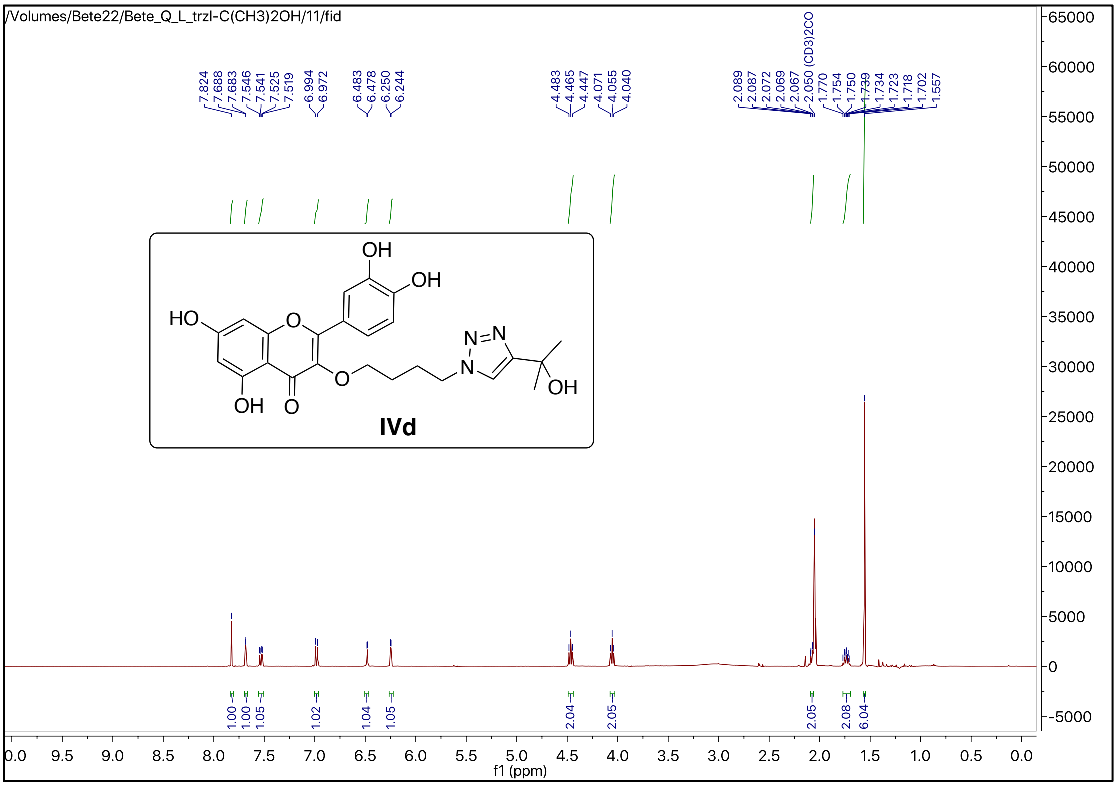

1.4.5. 2-(3,4-Dihydroxyphenyl)-5,7-dihydroxy-3-(4-(4-((2-isopropyl-5-methylphenoxy)methyl)-1H-1,2,3-triazol-1-yl)butoxy)-4H-chromen-4-one IVe; ^{1}H NMR (400 MHz, DMSO-d_6):

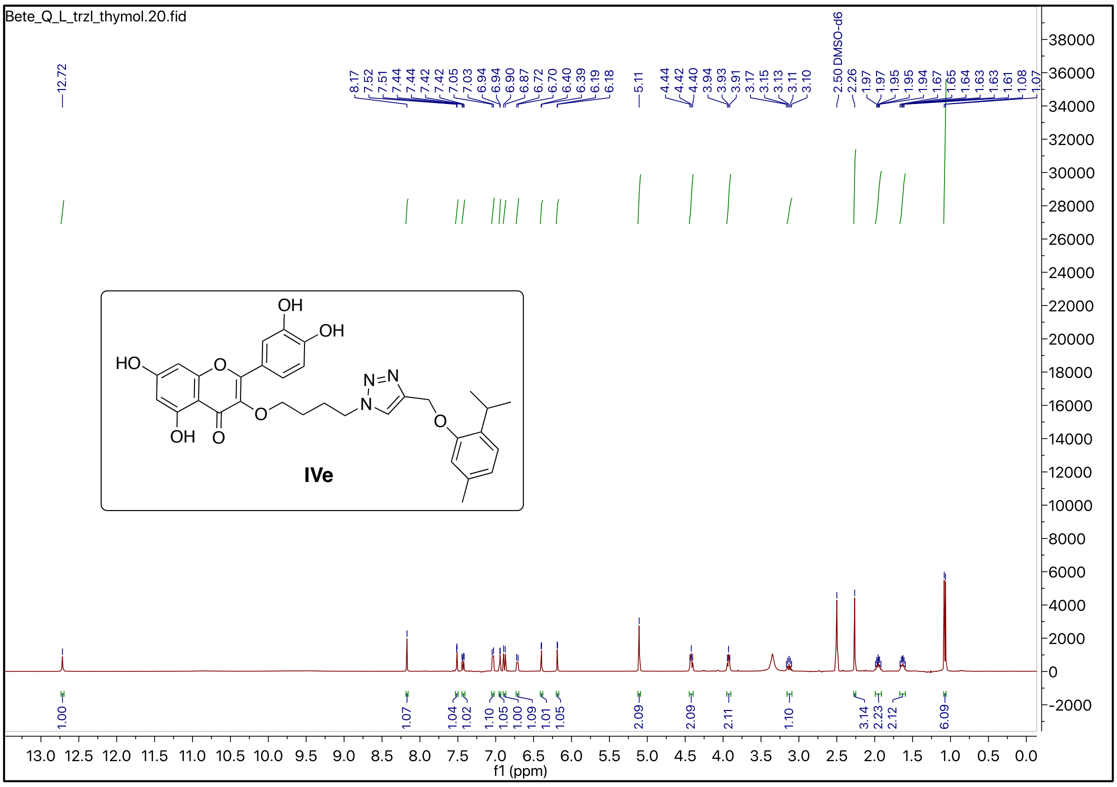

Supplement: Supplementary file 1 [file molecules-28-07495-s001.zip › molecules-2679547-supplementary.pdf]
